# Association of loneliness and social isolation with excess risk of mental disorders in people with obesity: a prospective cohort study

**DOI:** 10.1186/s12963-025-00451-4

**Published:** 2025-12-30

**Authors:** Ying Zhou, Rui Chen, Hongyu Yan, Xiaoxv Yin

**Affiliations:** 1 Department of Social Medicine and Health Management, School of Public Health, Tongji Medical College, Huazhong University of Science and Technology, No. 13 Hangkong Road, Wuhan, 430030 Hubei China; 2 Department of Geriatrics, Union Hospital, Tongji Medical College, Huazhong University of Science and Technology, Jiefang Avenue, Wuhan, 430022 Hubei China

**Keywords:** Loneliness, Social isolation, Mental disorders, Obesity, Cohort

## Abstract

**Background:**

Loneliness and social isolation are more prevalent among obese individuals. This study aims to explore the association of the level of loneliness and social isolation with the excess risk of mental disorders among obese people compared with non-obese people.

**Methods:**

A total of 219,086 participants (109,543 obese participants and 109,543 matched non-obese participants) from the UK Biobank were included at baseline. Loneliness and social isolation were assessed using a two-item scale and a three-item scale, respectively. Incident mental disorders, including substance use, psychotic disorders, mood disorders, depression, anxiety disorders, post-traumatic stress disorder, and behavioral syndromes, were ascertained through linkage to primary and secondary care records.

**Results:**

During a median (IQR) follow-up of 12.40 (11.50–13.20) years, a total of 18,280 obese participants developed mental disorders. Compared to the least lonely group, obese people in the moderate and most lonely groups had a significantly and progressively higher risk of mental disorders (moderately lonely: HR 1.37, 95% CI 1.33–1.42; most lonely: HR 1.73, 95% CI 1.65–1.81; *P*-trend < 0.001). We observed a similar pattern in terms of social isolation. The relative importance of loneliness and social isolation in predicting mental disorders ranked third and eighth among traditional risk factors. Compared to non-obese individuals, the excess risk of mental disorders for obese individuals varied considerably by levels of loneliness and social isolation, with HRs ranging from 1.05 in the least lonely to 1.79 in the most lonely, and from 1.14 in the least isolated to 1.36 in the most isolated.

**Conclusion:**

Alleviating loneliness and social isolation was associated with lower obesity-related excess risk of mental disorders. Our finding suggests the incorporation of social networking platforms and support systems into intervention strategies to effectively mitigate the mental health issues in obese population.

Clinical trial registration number: Not applicable. (This study is an observational analysis based on UK Biobank data and does not involve a clinical trial. This research was conducted under UK Biobank application no. 88159. The UK Biobank study was approved by the North West Multi-Centre Research Ethics Committee (11/NW/0382). All participants provided informed consent through electronic signature at baseline assessment.)

**Supplementary Information:**

The online version contains supplementary material available at 10.1186/s12963-025-00451-4.

## Introduction

Obesity affects over 890 million adults (16%) worldwide and has been declared a global epidemic by the World Health Organization [1]. Evidence shows that the risk of mental disorders in obese people ranges between 30% and 70%, significantly higher than in non-obese populations [2]. Mental disorders are one of the fastest-growing and heaviest public health threats and the leading causes of disability and death, accounting for 14% of the global disease burden [3, 4]. Given the high risk of mental disorders in individuals with obesity, identifying risk factors of mental disorders for them is essential.

Loneliness and social isolation are two important components of social determinants of health. Loneliness typically denotes negative subjective emotional feelings associated with the quality of social relationships [5], whereas social isolation is defined as an objective lack of quantity and frequency of interactions with friends, family, and the wider community [6]. Obese individuals may be particularly vulnerable to the mental health consequences of social disconnection due to a combination of psychosocial adversity and biological dysregulation. Persistent exposure to weight stigma could erode self-worth and prompt withdrawal from social interactions, thereby increasing both the risk of social isolation and the subjective experience of loneliness [7–9]. At the same time, obesity is associated with chronic low-grade inflammation, which may directly affect brain function by promoting neuroinflammatory processes linked to mood dysregulation. Concurrent alterations in stress physiology, including dysregulation of the hypothalamic–pituitary–adrenal (HPA) axis, may further impair emotional resilience, making individuals more susceptible to the psychological toll of social disconnection [10, 11]. Prior literature has extensively documented the importance of social contact in the etiology of mental health problems [11–15]. However, little focus has been placed on obese population.

This study aims to provide an answer to the question: What role do loneliness and social isolation play in mental health among people with obesity? Utilizing the UK Biobank cohort, we tested three specific hypotheses: First, that both loneliness and social isolation would be independently associated with an increased risk of mental disorders among people with obesity. Second, that loneliness and social isolation would demonstrate predictive strength comparable to traditional sociodemographic and lifestyle risk factors. Third, obese individuals with lower levels (versus higher levels) of loneliness/social isolation would be associated with a smaller excess risk of mental disorders relative to non-obese participants.

## Methods

### Study population

The UK Biobank is a large-scale prospective cohort study (https://www.ukbiobank.ac.uk/). Between 2006 and 2010, it recruited over 500,000 participants aged 40–70 years throughout England, Wales, and Scotland. Comprehensive data were collected via touchscreen questionnaires, verbal interviews, physical measurements, and biological samples. Further details regarding the design of the UK Biobank have been previously documented [16, 17].

Among a total of 502,401 participants at baseline, we excluded those with missing values for loneliness, social isolation, and obesity, and those with preexisting mental disorders. Participants with a body mass index (BMI; weight in kilograms divided by the square of height in meters) of 30 or greater were defined as obese. In the study of loneliness and social isolation with the risk of mental disorders among people with obesity, we included 109,543 obese participants. In the study of loneliness and social isolation with excess risk of mental disorders, we included 219,086 participants (109,543 obese participants were matched with an equal number of non-obese participants using Propensity Score Matching [PSM]). Propensity scores were estimated using logistic regression, adjusting for age (continuous), sex (categorical), and assessment center (categorical). The matching was performed using 1:1 nearest neighbor matching without replacement, with a caliper width of 0.2 standard deviations of the logit of the propensity score. The detailed flowchart is displayed in Supplementary Figure S1.

The UK Biobank study was approved by the North West Multi-Centre Research Ethics Committee. All participants provided informed consent through electronic signature at baseline assessment.

### Definition of loneliness and social isolation

We referred to previous studies to measure loneliness and social isolation in the UK Biobank database. Loneliness was measured through two questions in UK Biobank surveys: (1) “How often are you able to confide in someone close to you?” (“once a month”–“almost daily” was scored as 0 point, and “never/almost never” or “once every few months” was scored as 1 point) and (2) “Do you often feel lonely?” (“no” was scored as 0 point, and “yes” was scored as 1 point) [18]. Social isolation was measured using three questions: (1) “How often do you visit friends or family or have them visit you?” (“once a week”– “almost daily” was scored as 0 point, and “once a month” or less, or “no friends/family outside household” was scored as 1 point); (2) “Including yourself, how many people live in your household?” (living with one or more members was scored as 0 point, and living alone was scored as 1 point); (3) “Which of the following (leisure or social activities) do you engage in once a week or more often? (adult education class, pub or social club, sports club or gym, religious group, or other group activities)” (participating in one or more activities mentioned above was scored as 0 point, and not participating in any leisure or social activities was scored as 1 point) [6]. These items were selected based on their alignment with well-established scales. Two items of loneliness were modified from questions included in the validated revised UCLA Loneliness Scale [19, 20]. Three items of social isolation were drawn from similar questions in the validated Berkman–Syme social network index [21]. These items have demonstrated strong predictive validity in the UK Biobank cohort, showing significant associations with the risks of diabetes [6, 22], cardiovascular diseases [5], and mortality [23] in prior studies. The sum of the questionnaire items represented the index for loneliness (ranging from 0 to 2) and isolation (ranging from 0 to 3). Loneliness was categorized into three levels: index = 0, index = 1, and index = 2; isolation was also divided into three levels: index = 0, index = 1, and index ≥ 2 (Supplementary Table S1).

### Ascertainment of mental disorders

Diagnosis codes for all participants were derived from the UK Biobank Data-Field 41270. This field provides a comprehensive record of the International Classification of Disease version 10 (ICD-10) from hospital inpatient records, incorporating both primary and secondary diagnoses. The primary outcome of our study was all mental disorders (ICD-10: F00-F99). The secondary outcomes were substance use (ICD-10: F10-F19), psychotic disorders (including schizophrenia, schizotypal and delusional disorders, ICD-10: F20-F29), mood disorders (ICD-10: F30-F39), depression (ICD-10: F32-F33), anxiety disorders (ICD-10: F40-F48), post-traumatic stress disorder (PTSD, ICD-10: F43.1), and behavioral syndromes (ICD-10: F50-F59) [24–26]. The detailed ICD codes are shown in Supplementary Table S2. Follow-up duration was calculated starting from the baseline date until the date of first mental disorder diagnosis, death, end of follow-up (30 September 2021), or loss to follow-up, whichever occurred first.

### Covariates

We considered sociodemographic characteristics, lifestyle factors, and health conditions as confounding factors. Covariates included age (years), sex (male or female), ethnicity (white British or others), Townsend deprivation index (continuous; a higher score indicates lower socioeconomic status), educational attainment (college/university degree or other qualification), diet (healthy or unhealthy), smoking status (non-current smokers or current smokers), alcohol consumption (no heavy drinking or heavy drinking), exercise (adequate or inadequate), sleep duration (optimal or suboptimal), the number of chronic diseases (including cardiovascular disease, hypertension, type 2 diabetes, cancer, and respiratory disease; divided into three groups: 0, 1–2, ≥3). Lifestyle factors included modifiable behaviors that are reported to be linked to both social contact and mental health outcomes. A healthy diet was defined as an appropriate intake of at least five out of ten key dietary components (fruit, vegetable, fish, processed meats, unprocessed red meats, whole grains, refined grains, vegetable oils, dairy, sugar-sweetened beverages) [27, 28]. Smoking status was categorized as current or non-current. Alcohol consumption was calculated in grams, with targets set at ≤14 g/day for women and ≤28 g/day for men [29]. Sufficient physical activity was defined as ≥150 min of moderate-intensity or ≥75 min of vigorous-intensity activity per week. Adequate sleep duration was defined as 7–8 h/day [27]. Chronic diseases were ascertained through linkage to hospital inpatient records and self-reported information [30, 31]. More details on the assessment of lifestyles and chronic diseases are shown in Supplementary Tables S3–S5.

### Statistical analyses

Baseline characteristics of obese and non-obese participants, categorized by levels of loneliness and social isolation, were displayed as mean (standard deviation [SD]) for normally distributed continuous variables, median [interquartile range (IQR)] for nonnormally distributed continuous variables, and number (percentage) for categorical variables. Differences across groups were tested using ANOVA, Kruskal–Wallis test, and *χ*^2^ test, respectively.

Cox regression models were performed to explore the independent associations of loneliness and social isolation with the risk of all mental disorders, substance use, psychotic disorders, mood disorders, depression, anxiety disorders, PTSD, and behavioral syndromes, both in participants with and without obesity. The reference group was the group with an index of 0. Models were adjusted for age, sex, ethnicity, Townsend deprivation index, education, diet, smoking status, alcohol consumption, exercise, sleep duration, and the number of chronic diseases. We also categorized participants into four groups based on levels of loneliness and social isolation (low/low, high only in one, high in both) to explore the joint association of loneliness and social isolation with mental disorder risks.

To estimate the proportion of mental diseases in obese participants that hypothetically could be avoided by not feeling lonely or maintaining social connectedness (index = 0), we further categorized loneliness and social isolation into binary variables (loneliness: 0, 1–2; social isolation: 0, 1–3) and calculated the population attributable fraction (PAF). We used the coxphERR function in Rstudio to calculate the contribution of loneliness, social isolation, and other traditional risk factors to assess the relative importance of loneliness and social isolation among various risk factors in predicting mental disorders among people with obesity [5, 18].

In the subsequent analysis, we set the matched non-obese participants as the reference group to examine the excess risk of mental disorders in obese individuals at varying levels of loneliness and social isolation. The cumulative hazard of mental disorders corresponding to weight status and loneliness/social isolation index was presented.

We conducted subgroup and interaction analyses stratified by sociodemographic factors. Several sensitivity analyses were performed. First, we excluded people who developed mental disorders within 2 years from baseline to minimize reverse causation. Second, we treated death as a competitive event, and conducted competing risk analyses using Fine and Gray’s proportional sub-distribution hazards model. Third, we imputed missing data for loneliness and social isolation, and repeated our analyses.

All the statistical analyses were conducted using R version 4.3.1. Two-sided *P* < 0.05 indicates statistical significance.

## Results

### Baseline characteristics of participants

We included 109,543 obese participants (median [IQR] age: 51.0 [58.0, 63.0] years; 52.7% female) in this study. Among obese participants, 69,749 (63.67%), 31,135 (28.42%), and 8659 (7.9%) were defined with a loneliness index of 0, 1, and 2; and 46,673 (42.61%), 45,492 (41.53%), and 17,378 (15.86%) were defined with a social isolation index of 0, 1, and ≥2, respectively (Table 1). The characteristics of non-obese participants are shown in Supplementary Table S6. The prevalence of both loneliness and social isolation was significantly higher in the obese population than in the non-obese population (*P* < 0.001) (Supplementary Table S7).

**Table 1:** Baseline characteristics of obese participants

	Overall (*N* = 109,543)	Loneliness				Social isolation			
		Index = 0 (*N* = 69,749)	Index = 1 (*N* = 31,135)	Index = 2 (*N* = 8659)	*P* value	Index = 0 (*N* = 46,673)	Index = 1 (*N* = 45,492)	Index ≥ 2 (*N* = 17,378)	*P* value
Age (years)	51.0 [58.0, 63.0]	58.0 [51.0, 63.0]	58.0 [51.0, 63.0]	57.0 [50.0, 62.0]	<0.001	59.0 [51.0, 64.0]	58.0 [51.0, 63.0]	57.0 [51.0, 63.0]	<0.001
Female	57,676 (52.7%)	36,392 (52.2%)	16,635 (53.4%)	4649 (53.7%)	<0.001	24,017 (51.5%)	24,700 (54.3%)	8959 (51.6%)	<0.001
White British	97,698 (89.2%)	63,019 (90.4%)	27,183 (87.3%)	7496 (86.6%)	<0.001	42,219 (90.5%)	40,428 (88.9%)	15,051 (86.6%)	<0.001
Townsend deprivation index	−3.4 [−1.8, 1.1]	−2.1 [−3.6, 0.6]	−1.4 [−3.2, 1.8]	−0.7 [−2.9, 2.5]	<0.001	−2.2 [−3.6, 0.3]	−1.6 [−3.3, 1.3]	−0.6 [−2.9, 2.6]	<0.001
College/university degree	27,854 (25.4%)	19,021 (27.3%)	7050 (22.6%)	1783 (20.6%)	<0.001	12,011 (25.7%)	11,311 (24.9%)	4532 (26.1%)	<0.001
Healthy diet	16,879 (15.4%)	10,868 (15.6%)	4733 (15.2%)	1278 (14.8%)	0.066	7106 (15.2%)	7032 (15.5%)	2741 (15.8%)	0.217
No current smoking	99,438 (90.8%)	63,978 (91.7%)	27,934 (89.7%)	7526 (86.9%)	<0.001	43,168 (92.5%)	41,151 (90.5%)	15,119 (87.0%)	<0.001
Moderate alcohol consumption	81,551 (74.4%)	51,432 (73.7%)	23,471 (75.4%)	6648 (76.8%)	<0.001	32,683 (70.0%)	34,637 (76.1%)	14,231 (81.9%)	<0.001
Sufficient physical activity	52,458 (47.9%)	34,194 (49.0%)	14,459 (46.4%)	3805 (43.9%)	<0.001	24,508 (52.5%)	21,080 (46.3%)	6870 (39.5%)	<0.001
Healthy sleep duration	68,192 (62.3%)	45,855 (65.7%)	17,977 (57.7%)	4360 (50.4%)	<0.001	30,373 (65.1%)	28,023 (61.6%)	9796 (56.4%)	<0.001
The number of chronic diseases					<0.001				<0.001
0	49,591 (45.3%)	32,452 (46.5%)	13,662 (43.9%)	3477 (40.2%)		21,877 (46.9%)	20,259 (44.5%)	7455 (42.9%)	
1–2	55,335 (50.5%)	34,800 (49.9%)	15,939 (51.2%)	4596 (53.1%)		23,113 (49.5%)	23,285 (51.2%)	8937 (51.4%)	
≥3	4617 (4.2%)	2497 (3.6%)	1534 (4.9%)	586 (6.8%)		1683 (3.6%)	1948 (4.3%)	986 (5.7%)	

Data are *N* (%) for categorical variables, mean (SD) for normally distributed continuous variables, and median [IQR] for nonnormally distributed continuous variables

### Loneliness and social isolation with the risk of mental disorders among participants with obesity

During a median (IQR) follow-up of 12.40 (11.50–13.20) years, a total of 18,280 obese participants developed one or more mental disorders, which included 6952 cases of substance use disorders, 261 cases of psychotic disorders, 7678 cases of mood disorders, 7512 cases of depression, 5628 cases of anxiety disorders, 128 cases of PTSD, and 201 cases of behavioral syndromes.

**Fig. 1:**
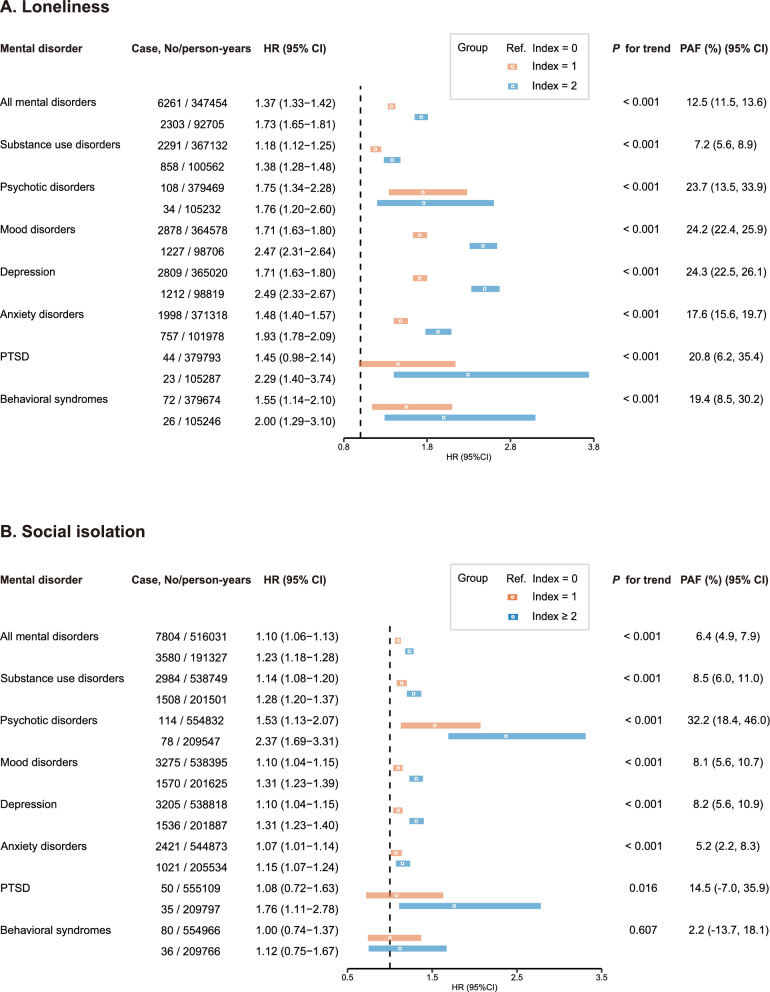
Association of loneliness (**A**) and social isolation (**B**) with risk of mental disorders among participants with obesity. CI, confidence interval; HR, hazard ratio; PAF: population attributed fraction; PTSD: post-traumatic stress disorder. Adjusted for age, sex, ethnicity, Townsend deprivation index, education, diet, smoking status, alcohol consumption, exercise, sleep duration, and the number of chronic diseases

Compared to the least lonely group, people in the moderate and most lonely groups had a significantly and progressively higher risk of mental disorders in the multivariate-adjusted model (moderately lonely: HR 1.37, 95% CI 1.33–1.42; most lonely: HR 1.73, 95% CI 1.65–1.81; *P*-trend < 0.001) (Fig. 1). Similarly, we observed a progressive increase in the risk of mental disorders with increasing levels of social isolation (moderately isolated: HR 1.10, 95% CI 1.06–1.13; most isolated: HR 1.23, 95% CI 1.18–1.28; *P*-trend < 0.001). It was estimated that, in people with obesity, the PAF for mental disorders related to loneliness was 12.5% (95% CI: 11.5–13.6%), and the PAF for mental disorders related to social isolation was 6.4% (95% CI: 4.9–7.9%).

We further analyzed the subtypes of mental disorders and found that the risk of substance use disorders, psychotic disorders, mood disorders, depression, and anxiety disorders all increased significantly with the level of loneliness/social isolation. For PTSD, only the highest degree of loneliness/social isolation elevated the risk of incidence. For behavioral syndrome, increasing levels of loneliness were associated with a gradual increase in its risk, whereas social isolation did not have a significant impact on its risk. We also performed the same analysis in the non-obese population, which is shown in Supplementary Figure S2. The associations of individual indicators of loneliness and social isolation with the risk of mental disorders were analyzed (Supplementary Table S8). Among people with obesity, most of the individual indicators of loneliness and social isolation were significantly associated with mental disorders. Supplementary Table S9 presents the joint exposure of loneliness and social isolation with the risk of mental disorders among obese people. Compared to those neither lonely nor isolated, people with social isolation alone showed a significantly elevated risk (fully adjusted HR 1.06, 95% CI 1.02–1.10), those with loneliness alone exhibited a stronger association (HR 1.40, 95% CI 1.33–1.47), while the highest risk was observed in those with both loneliness and social isolation (HR 1.55, 95% CI 1.49–1.61).

### Relative importance of loneliness and social isolation compared with traditional risk factors in predicting mental disorders among obese participants

**Fig. 2:**
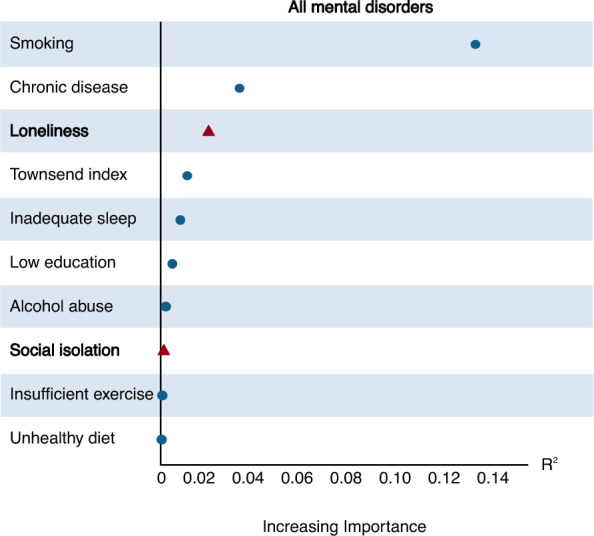
The relative importance of loneliness and social isolation comparing with other traditional risk factors in predicting all mental disorders in obese people

The relative importance of loneliness and social isolation compared with traditional risk factors (education, socioeconomic status, smoking, drinking, sleeping, exercising, diet, and chronic disease) in predicting mental disorders in obese people was calculated. Among selected risk factors, loneliness and social isolation ranked third and eighth in predicting all kinds of mental disorders among people with obesity, respectively (Fig. 2). Loneliness was among the top three risk factors in predicting psychotic disorders, mood disorders, depression, anxiety disorders, PTSD, and behavioral syndromes. Social isolation was the third important risk factor in predicting psychotic disorders (Supplementary Figure S3).

### Loneliness and social isolation with excess risk of mental disorders among obese people compared with non-obese people

**Fig. 3:**
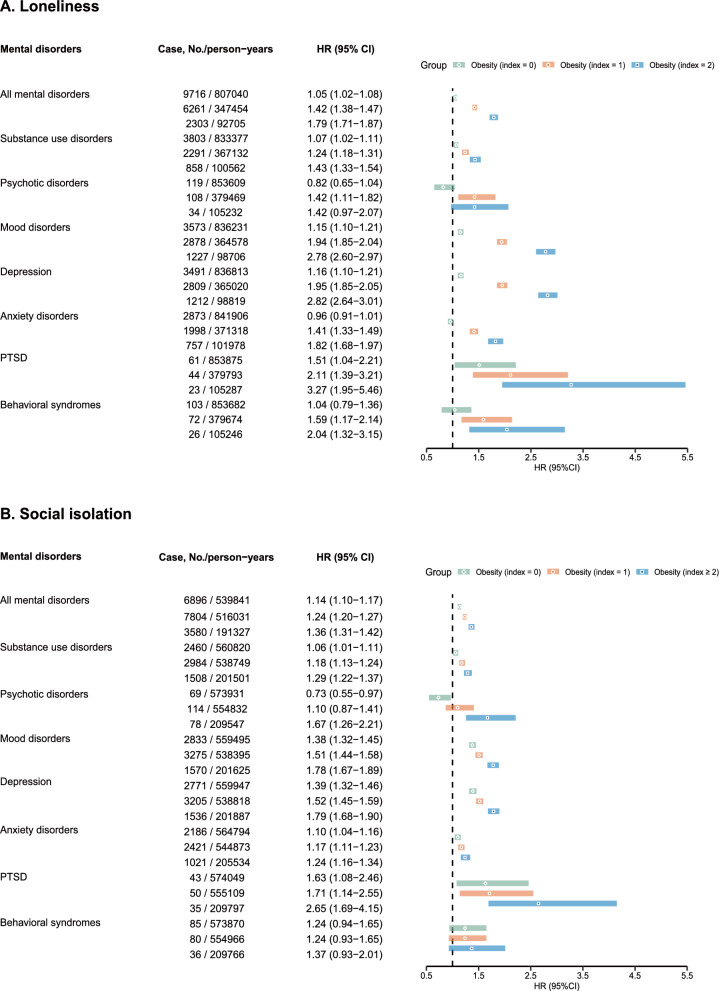
Association of loneliness (**A**) and social isolation (**B**) with excess risk of mental disorders among obese people compared with non-obese people. CI, Confidence interval; HR, Hazard ratio; PTSD: post-traumatic stress disorder. Adjusted for age, sex, ethnicity, Townsend deprivation index, education, diet, smoking status, alcohol consumption, exercise, sleep duration, and the number of chronic diseases

**Fig. 4:**
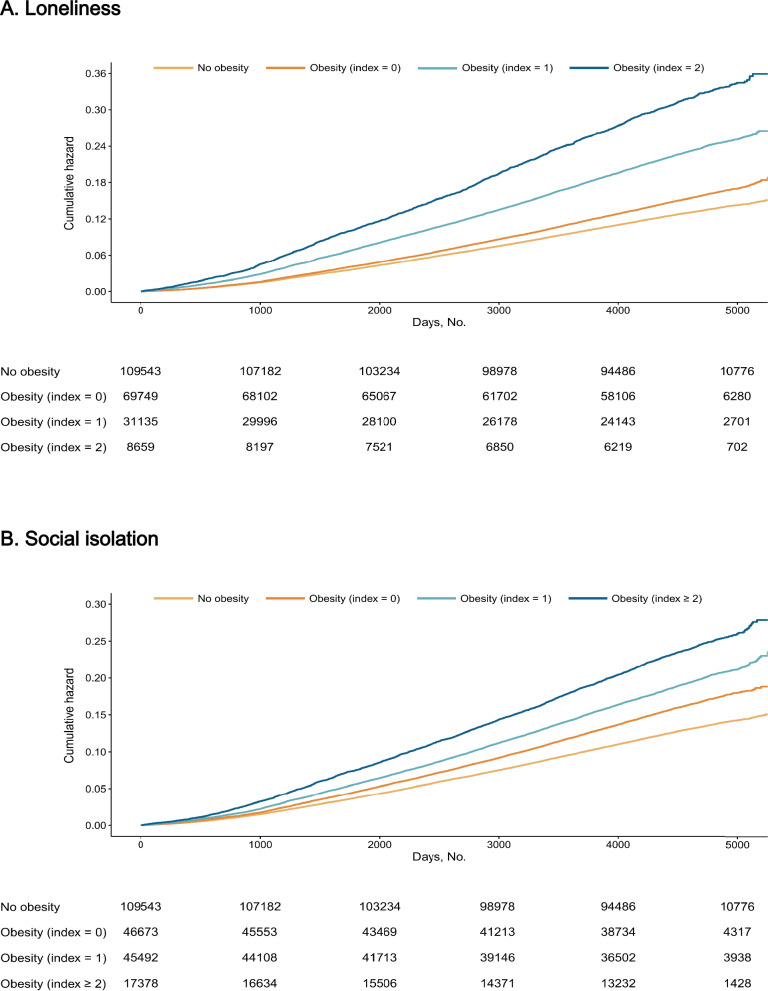
Cumulative hazard of mental disorders by weight status and loneliness (**A**)/social isolation (**B**) index

After PSM, acceptable between-group balance was achieved, with standardized mean differences of 0.031 for age, 0.014 for sex, and 0.117 for assessment centre (Supplementary Table S10). We evaluated the excess risk of mental disorders in obese individuals at different levels of loneliness and social isolation, compared to a matched non-obese group. As illustrated in Fig. 3, the HR for mental disorders among individuals with obesity varied substantially according to baseline loneliness/social isolation level. Compared to non-obese individuals, those with obesity and the lowest level of loneliness had a slightly elevated risk (HR 1.05, 95% CI 1.02–1.08). In contrast, those with medium and high loneliness levels exhibited progressively higher risks (HR 1.42 [1.38–1.47] and HR 1.79 [1.71–1.87], respectively). A similar pattern was observed for social isolation: obese individuals with the lowest level showed a modest increase in risk (HR 1.14, 95% CI 1.10–1.17), while those with medium and high levels had incrementally higher risks (HR 1.24 [1.20–1.27] and HR 1.36 [1.31–1.42], respectively). The cumulative hazard of mental disorders corresponding to weight status and loneliness/social isolation index is presented in Fig. 4 (cumulative hazards for subtypes of mental disorders are shown in Supplementary Figures S4–S5).

### Subgroup and sensitivity analysis

The results of subgroup analyses stratified by sociodemographic characteristics are shown in Supplementary Tables S11–S12. Significant interactions were observed between loneliness and age (*P* interaction < 0.001), and between social isolation and Townsend deprivation index (*P* interaction = 0.001) on the risk of all mental disorders. Specifically, the association between loneliness and the risk of mental disorders was stronger in obese individuals under 60 years of age (under 60 years: HR 1.88, 95% CI 1.77–2.00; 60 years and above: HR 1.60, 95% CI 1.49–1.72). Among obese individuals with lower socioeconomic status, the association between social isolation and the risk of mental disorders was more pronounced (low socioeconomic status: HR 1.33, 95% CI 1.26–1.40; high socioeconomic status: HR 1.16, 95% CI 1.09–1.25). Our results remained robust in the sensitivity analyses that excluded patients who developed mental disorders within 2 years from baseline, considered the competing risk event, and imputed exposure data (Supplementary Tables S13–S15).

## Discussion

In this large prospective cohort study, we found that loneliness and social isolation were associated with higher risk of a wide range of mental disorders in obese population. Loneliness and social isolation ranked third and eighth, respectively, in predicting mental disorders among traditional risk factors. Compared to non-obese individuals, the excess risk of mental disorders for obese individuals varied considerably by levels of loneliness and social isolation, with HRs ranging from 1.05 in the least lonely to 1.79 in the most lonely, and from 1.14 in the least isolated to 1.36 in the most isolated.

We found significant associations of loneliness and social isolation with a range of mental disorders among obese population. The results of our study were consistent with previous research conducted in general population, where loneliness and isolation were proven to be associated with higher risk of mental disorders. An in-person interview survey included 1700 participants from South Korea in 2019, and showed that loneliness and social isolation were associated with depression, social anxiety symptoms, and even suicide [32]. A cross-sectional study used a nationally representative sample of 34,653 American adults in 2004–2005, and demonstrated that the lack of close friends was positively related to generalized anxiety disorder, major depressive disorder, social phobia, and dysthymic disorder; the lack of frequently contacted religious group members was associated with increased risks of alcohol abuse and dependence, nicotine dependence, and drug abuse [33]. Our study extended this finding to obese individuals, a group with higher levels of loneliness and social isolation, and included a wider range of mental disorders as study outcomes.

Additionally, when comparing the relative importance of multiple common risk factors for mental disorders, we found that loneliness ranked third in predicting all mental illnesses, only behind smoking and the number of chronic diseases. In the case of mood disorders, depression, and anxiety disorders, loneliness was even the top predictive factor, with relative importance far exceeding that of other risk factors (including the number of chronic diseases, inadequate sleep, smoking, low education, insufficient exercise, alcohol abuse, unhealthy diet, social isolation, and Townsend deprivation index). This indicated the significant role that loneliness played in estimating mental disorders in obese patients. Social isolation ranked eighth in predicting all mental disorders, inferior to loneliness. Although loneliness and social isolation may have some conceptual overlap, they also have significant distinctions [34, 35]. Loneliness refers to the subjective feeling of a lack of connection or companionship, which is a psychological state. Social isolation, on the other hand, refers to the lack of objective frequency of social interactions. Social isolation does not necessarily imply loneliness; it may also be a matter of personal preference for some individuals. Consequently, loneliness tends to exert more psychological pressure on obese individuals than social isolation. This may help explain the differing associations of loneliness and social isolation with mental disorders.

The associations observed in this study can be explained through interconnected biopsychosocial mechanisms. Biologically, both obesity and loneliness/social isolation can promote a state of chronic low-grade inflammation and dysregulate the HPA axis [10], which governs stress responses. Their co-occurrence may disrupt brain function, thereby increasing vulnerability to mental disorders. Psychologically, loneliness can foster negative thought patterns and impair emotional regulation, which may contribute to the development of mental disorders. Concurrently, the distress from loneliness and weight stigma can overwhelm coping capacity, prompting maladaptive strategies like emotional eating. This not only worsens physical health but also deepens psychological distress. From a socio-behavioral perspective, loneliness and social isolation often limit access to supportive resources and are linked to unhealthy behaviors like reduced physical activity and sleep disturbances [11], collectively forming a pathway from social disconnection to mental disorders.

Our subgroup analyses revealed significant effect modifications by sociodemographic factors. First, we observed a stronger association between loneliness and mental disorder risk among obese individuals under 60 years. This pattern may reflect age-dependent differences in social needs and emotion regulation capacity, consistent with the Socioemotional Selectivity Theory proposed by Carstensen [36]. Middle-aged adulthood represents critical periods for establishing careers, families, and core social networks. Individuals in these life stages typically place greater emphasis on social integration, identity formation, and interpersonal connections. Consequently, experiences of loneliness may be perceived as more salient signals of “social failure.” In contrast, older adults have often undergone more social role transitions and may possess greater emotional resilience. Having developed more adaptive coping strategies over their lifespan, they are potentially better equipped to buffer the detrimental effects of loneliness. Additionally, the association between social isolation and mental disorder risk was more pronounced among obese individuals with lower socioeconomic status. This finding may be interpreted through the lens of the Resource Substitution Hypothesis [37]. Individuals with higher socioeconomic status can draw upon substantial material and cultural resources to partially compensate for the negative impacts of social isolation. For example, they have greater access to high-quality solitary activities (such as fitness training, online courses, and cultural consumption) that can still provide a sense of accomplishment and psychological sustenance. Conversely, individuals with lower socioeconomic status often rely more heavily on free or low-cost leisure activities—such as community gatherings and use of public spaces—that are inherently social in nature. When confronted with social isolation, these individuals not only lack meaningful alternative solitary pursuits but also face the compounded burdens of economic strain and social exclusion, leading to a magnified mental health risk.

Furthermore, using non-obese individuals as the reference group, we analyzed the excess risk of mental disorders among obese individuals with varying degrees of loneliness and social isolation. We found that, compared to non-obese individuals, the excess risk of mental disorders for obese individuals varied considerably by levels of loneliness and social isolation, with HRs ranging from 1.05 in the least lonely to 1.79 in the most lonely, and from 1.14 in the least isolated to 1.36 in the most isolated. Given the high prevalence of obesity globally, the significant reduction in the percentage of this risk could lead to a considerable decrease in the number of patients suffering from mental disorders, and thus has significant public health implications. This suggests the incorporation of social networking platforms and support systems into community intervention strategies to enhance social connections and reduce lonely feelings in the obese population, thereby effectively mitigating their risk of mental health issues.

Our findings demonstrate that loneliness and social isolation are significant risk factors for mental disorders in people with obesity. Of particular concern is that the psychosocial burden associated with obesity may be linked to more severe mental health outcomes, including suicidal risk. For instance, a recent study of women with fibromyalgia and obesity found that psychological factors, such as depressive symptomatology and pain catastrophizing, were significantly associated with suicidal ideation [38]. This evidence suggests that the “psychosocial pain” stemming from the interplay of loneliness, social isolation, and weight stigma may be analogous to chronic physical pain in its capacity to deplete an individual’s psychological resources, thereby potentially leading to extreme outcomes like suicidal ideation. Consequently, the pathway from loneliness and isolation to mental disorders that we have identified may represent a critical segment of a trajectory toward more severe clinical endpoints. This perspective underscores the importance of early intervention for this vulnerable population.

To the best of our knowledge, this study might be the largest and most comprehensive study to date on the association between social contact and a wide range of mental disorders in obese population. The large sample size and long follow-up time provided sufficient outcome events and adequate statistical explanatory power for our results. However, limitations also need to be declared. First, loneliness and social isolation were assessed at baseline, and we were unable to capture their dynamic changes over time. This reliance on a single baseline measurement likely resulted in non-differential misclassification of exposure status during the follow-up period. Such misclassification would generally bias the observed HRs toward the null. Consequently, the true associations are likely stronger than those reported in our present analyses. Second, the ascertainment of mental disorders relied on hospital records, which may lead to diagnostic undercapture (e.g., individuals with subthreshold symptoms or without clinical contact). This under-ascertainment may be more pronounced in those with obesity, loneliness, or social isolation due to greater healthcare access barriers. If this bias exists, this detection bias would attenuate the observed association, making our findings likely conservative. Third, this study used brief measures for loneliness and social isolation. While these indices are informed by established scales and have demonstrated predictive validity in UK Biobank, they are simplified proxies that may not capture the full complexity of multidimensional scales. Fourth, limited by the nature of observational studies, we were unable to make causal inferences. A particular concern is the potential for reverse causality. Although we excluded participants with pre-existing mental disorders at baseline, it is possible that undiagnosed prodromal symptoms of emergent mental illness could have influenced the self-reporting of loneliness at baseline. Such individuals might experience social withdrawal and negative affect as early manifestations of their condition, which would be measured as loneliness in our study. Notably, our subgroup analysis provides some support for this possibility. We found that the association between loneliness and incident mental disorders was significantly stronger in younger participants. This pattern is consistent with the hypothesis that in younger populations, loneliness may itself be an early indicator of a developing mental disorder. In contrast, we observed no significant effect modification by age for the association between social isolation and mental disorders. This difference is informative. Social isolation, as an objective measure of social network, is likely to be a more stable risk factor. Future studies with repeated measures of both psychosocial exposures and mental health symptoms are needed to clarify these complex relationships. Fifth, although we employed rigorous methods including PSM and comprehensive covariate adjustment, residual confounding from unmeasured factors (e.g., health literacy, personality traits, or quality of social support) could not be ruled out, which is an inherent limitation of observational cohort studies [39]. Finally, approximately 90% of our study participants were White British. Considering the differences among various racial groups in terms of physiological characteristics, cultural environments, and behavioral patterns, caution should be exercised when generalizing our findings to other racial or ethnic groups.

## Conclusions

In this study, loneliness and social isolation were associated with increased risk of a broad range of mental disorders among obese individuals. Loneliness ranked third among the traditional predictors of mental disorders. Our findings highlight the importance of controlling loneliness and social isolation in decreasing the risk of mental disorders in individuals living with obesity.

## Supplementary Information

Additional file 1

## Data Availability

Data from UK Biobank are available on application at www.ukbiobank.ac.uk/register-apply.

## Abbreviations

BMI: Body mass index
HPA: Hypothalamic–pituitary–adrenal
ICD-10: International Classification of Disease version 10
SD: Standard deviation
IQR: Interquartile range
PTSD: Post-traumatic stress disorder
PAF: Population attributable fraction
PSM: Propensity score matching
