# Supplementary material for: Association of loneliness and social isolation with excess risk of mental disorders in people with obesity: a prospective cohort study
Source: Popul Health Metr. 2025 Dec 30;24:9. doi: 10.1186/s12963-025-00451-4 (PMC12859870; doi:10.1186/s12963-025-00451-4)
Supplement: Supplementary file 1 — Additional file 1 [file 12963_2025_451_MOESM1_ESM.docx]

**Association of loneliness and social isolation with excess risk of mental disorders in people with obesity: a prospective cohort study**

**Appendix:**

[Figure S1. Flow chart of the inclusion of participants 3](#_Toc213527725)

[Figure S2. Associations of loneliness (A) and social isolation (B) with risk of mental disorders in the non-obese participants 4](#_Toc213527726)

[Figure S3. The relative importance of loneliness and social isolation compared with other traditional risk factors in predicting different mental disorders in obese people. 6](#_Toc213527727)

[Figure S4. The cumulative hazard of subtypes of mental disorders corresponding to weight status and loneliness index. 9](#_Toc213527728)

[Figure S5. The cumulative hazard of subtypes of mental disorders corresponding to weight status and social isolation index. 12](#_Toc213527729)

[Table S1. Definition of loneliness and social isolation in the UK Biobank 13](#_Toc213527730)

[Table S2. Definition of mental disorders in the UK Biobank 14](#_Toc213527731)

[Table S3. Definitions of lifestyles in the UK Biobank 15](#_Toc213527732)

[Table S4. Components of diet quality score used in the UK Biobank 16](#_Toc213527733)

[Table S5. Definitions of chronic diseases in the UK Biobank 18](#_Toc213527734)

[Table S6. Baseline characteristics of non-obese participants 19](#_Toc213527735)

[Table S7. Distribution of loneliness and social isolation in the obese and non-obese participants 20](#_Toc213527736)

[Table S8. Associations of individual indicator of loneliness and social isolation with risk of mental disorders in the obese participants 21](#_Toc213527737)

[Table S9. Associations of joint exposure of loneliness and social isolation with risk of mental disorders in the obese participants 25](#_Toc213527738)

[Table S10. The standardized mean differencess of age, sex, and assessment center before and after propensity score matching 27](#_Toc213527739)

[Table S11. Subgroup analyses of the associations between loneliness and the risk of mental disorders in the obese participants stratified by sociodemographic characteristics 28](#_Toc213527740)

[Table S12. Subgroup analyses of the associations between social isolation and the risk of mental disorders in the obese participants stratified by sociodemographic characteristics 32](#_Toc213527741)

[Table S13. Associations of loneliness and social isolation with risk of mental disorders in the obese participants after excluding patients who developed mental disorders within two years from baseline 36](#_Toc213527742)

[Table S14. Associations of loneliness and social isolation with risk of mental disorders in the obese participants considering competing risk event 39](#_Toc213527743)

[Table S15. Associations of loneliness and social isolation with risk of mental disorders in the obese participants with the imputation of exposure data 42](#_Toc213527744)

**Exclusion:**

- Without data for exposure (n = 35 710)
- Without data for obesity (n = 2 227)
- With mental disorders at baseline

(n = 10 015)

Participants accessed for eligibility (n = 454 449)

1:1 matched on age, sex, and center (n = 219 086)

Participants from the UK biobank (n = 502 401)

Without obesity at baseline (n = 344 906)

With obesity at baseline

(n = 109 543)

**Exclusion:**

- Underweight (BMI < 18.5 kg/m^2^) (n = 2 238)
- Overweight (BMI: 25.0~29.9 kg/m^2^) (n = 193 634)
- Unmatched (n = 39 491)

Matched cohort analysis

(n = 219 086)

No obesity cohort

(n = 109 543)

Obesity cohort

(n = 109 543)

Figure S1. Flow chart of the inclusion of participants

**A. Loneliness**

**
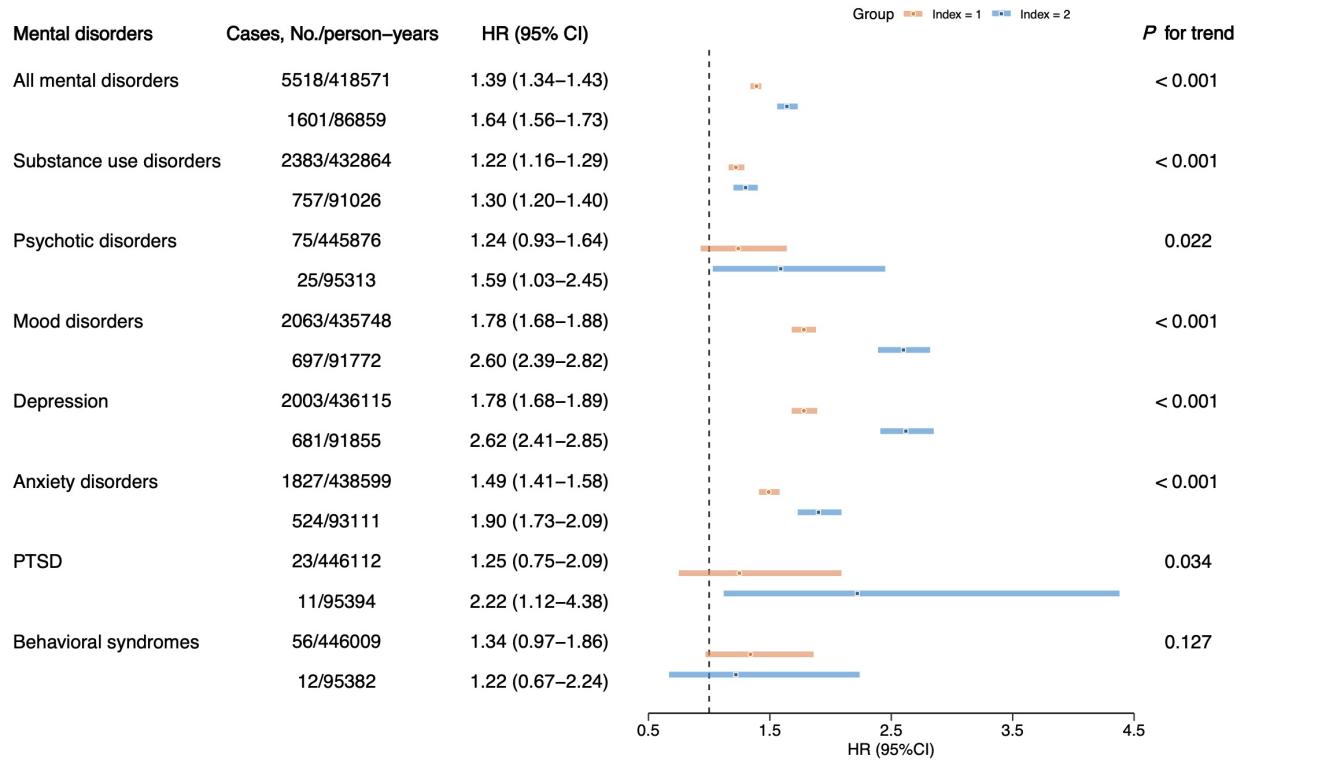
**

**B. Social isolation**

**
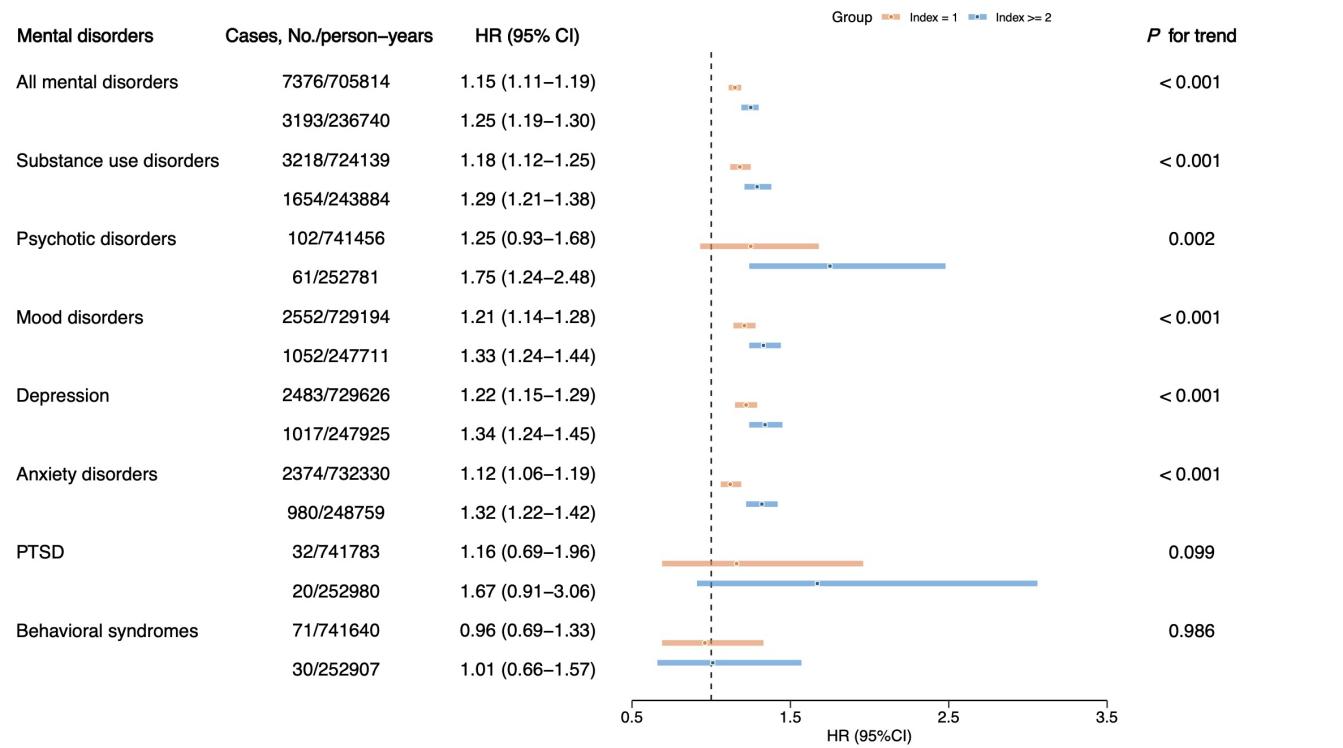
**

Figure S2. Associations of loneliness (A) and social isolation (B) with risk of mental disorders in the non-obese participants

Abbreviations: CI, Confidence interval; HR, Hazard ratio; PTSD: post-traumatic stress disorder. Adjusted for age, sex, ethnicity, Townsend deprivation index, education, diet, smoking status, alcohol consumption, exercise, sleep duration, and the number of chronic diseases.

**A. Substance use disorders B. Psychotic disorders**


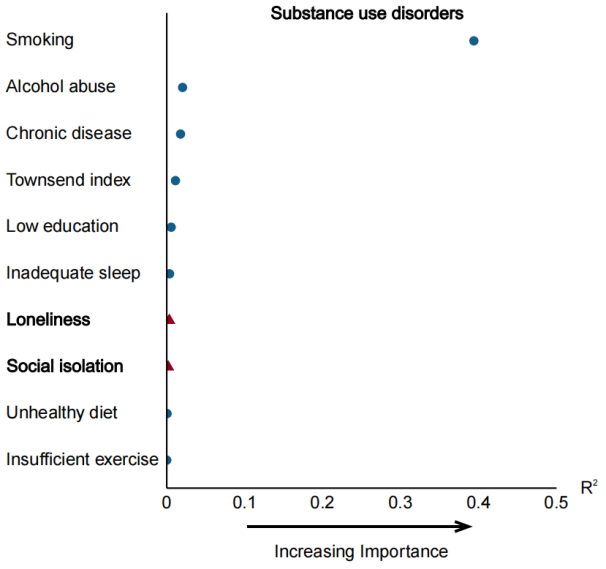

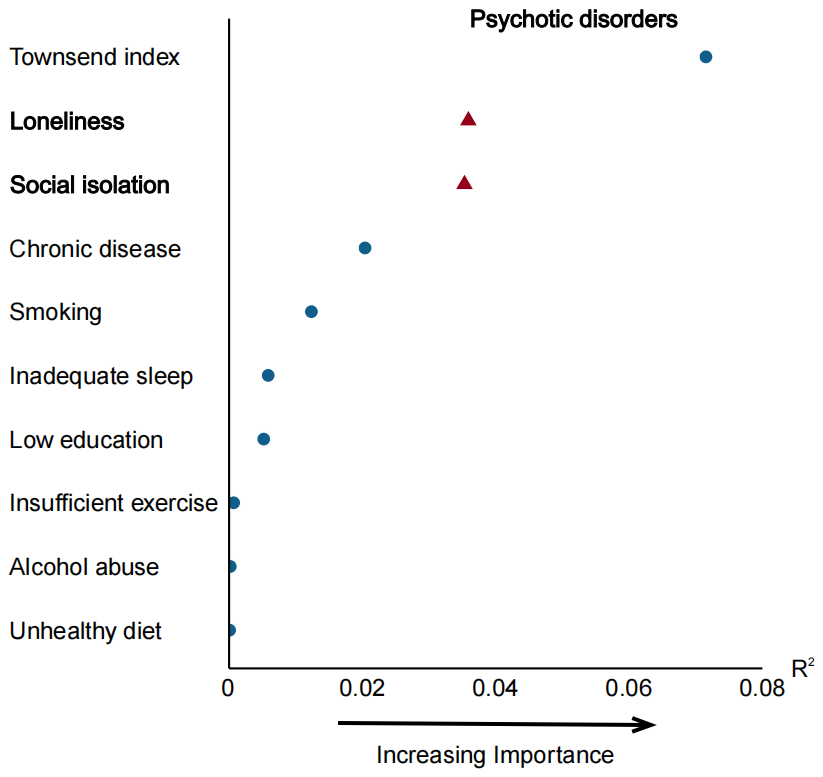


**C. Mood disorders D. Depression**


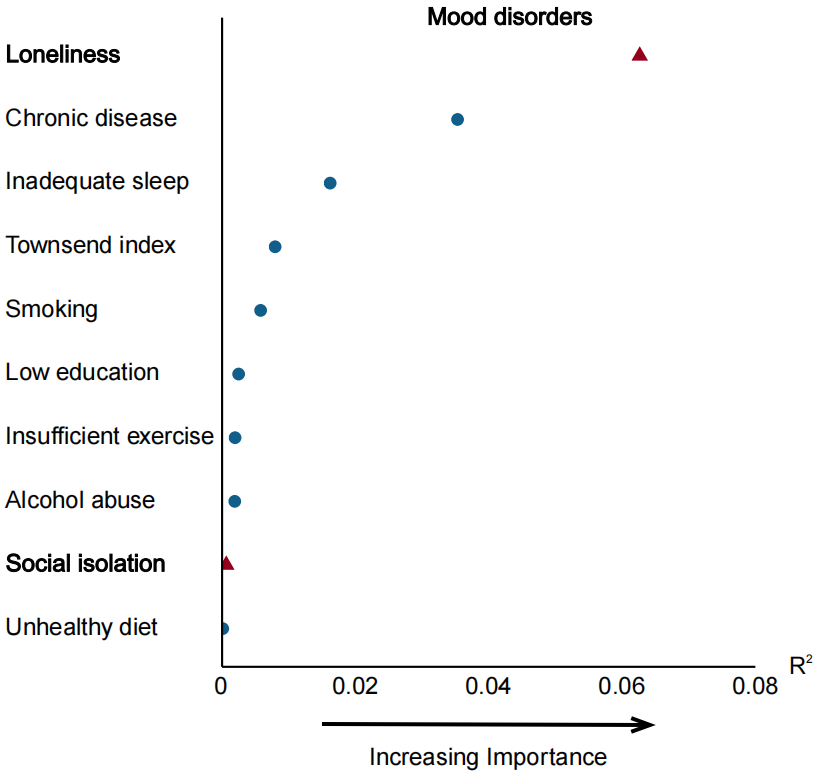

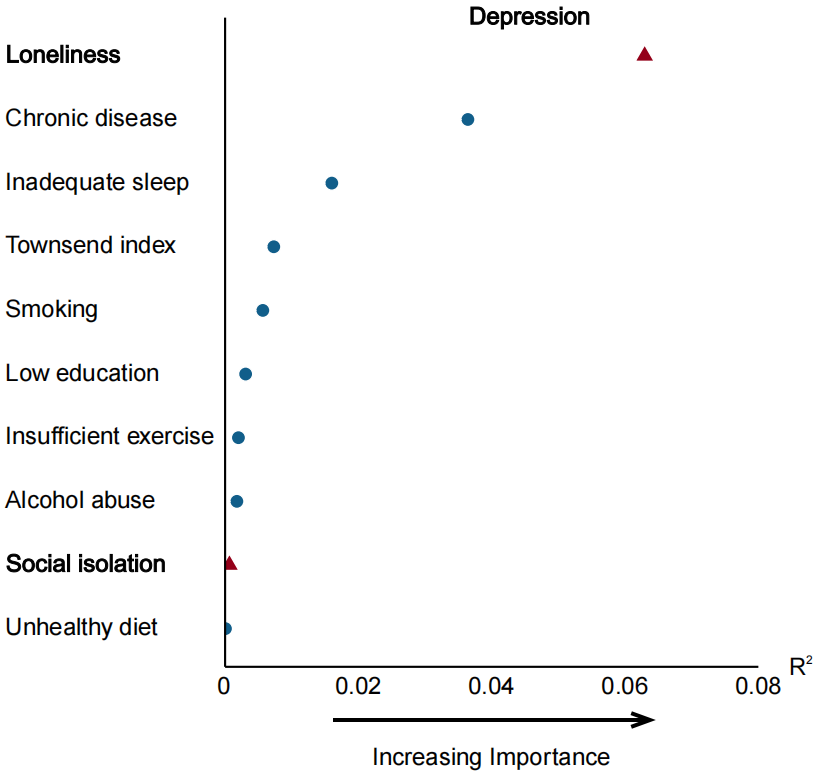


**E. Anxiety disorders F. PTSD**


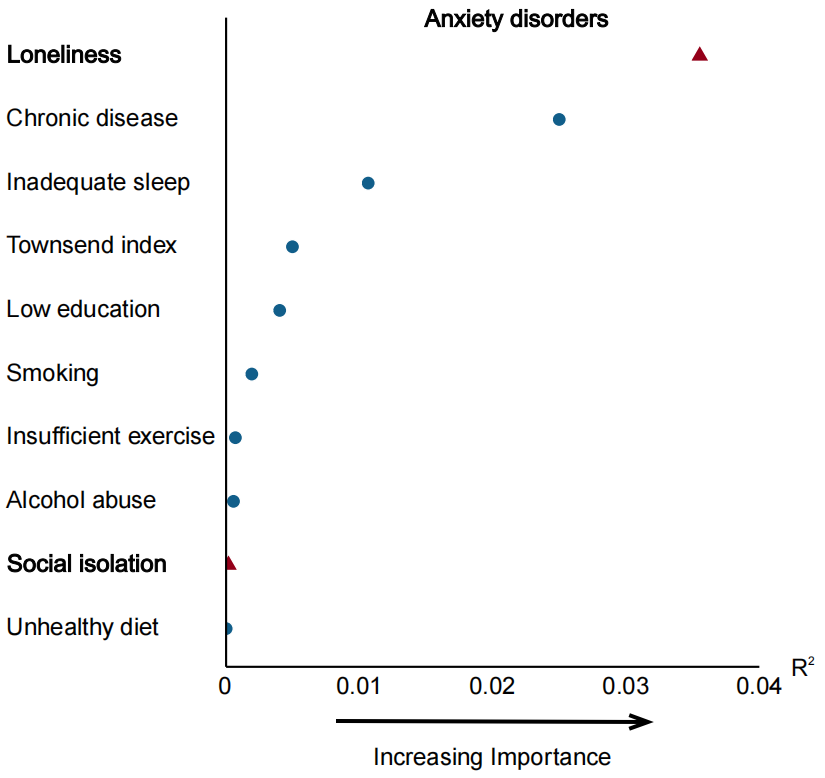

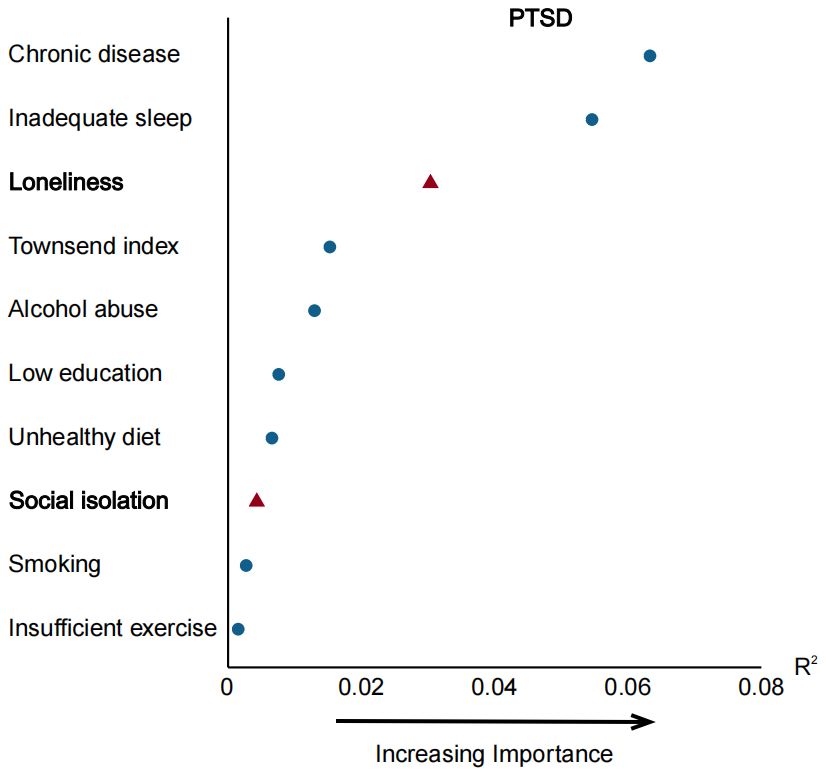


**G. Behavioral syndromes**

**
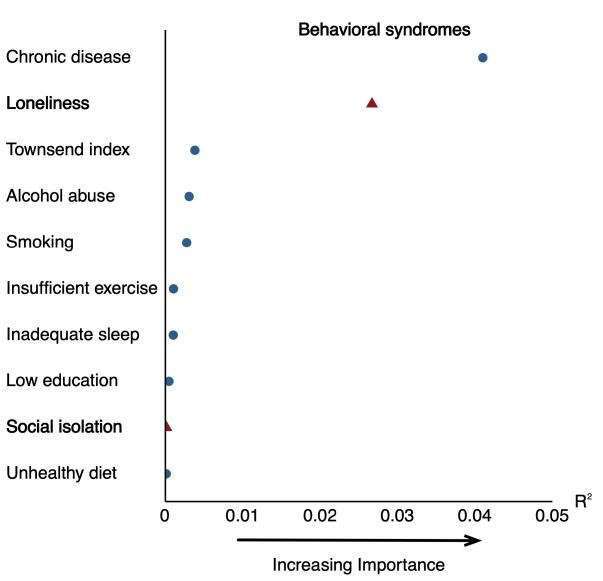
**

Figure S3. The relative importance of loneliness and social isolation compared with other traditional risk factors in predicting different mental disorders in obese people.

(A) Substance use disorders; (B) Psychotic disorders; (C) Mood disorders; (D) Depression; (E) Anxiety disorders; (F) PTSD; (G) Behavioral syndromes.

Abbreviations: PTSD: post-traumatic stress disorder.

**A. Substance use disorders**


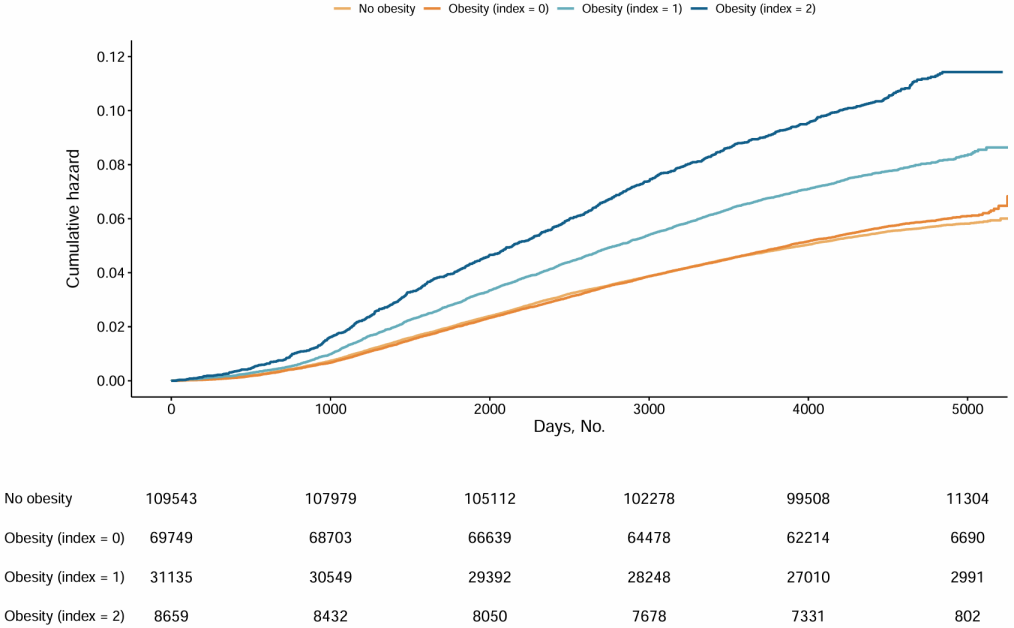


**B. Psychotic disorders**


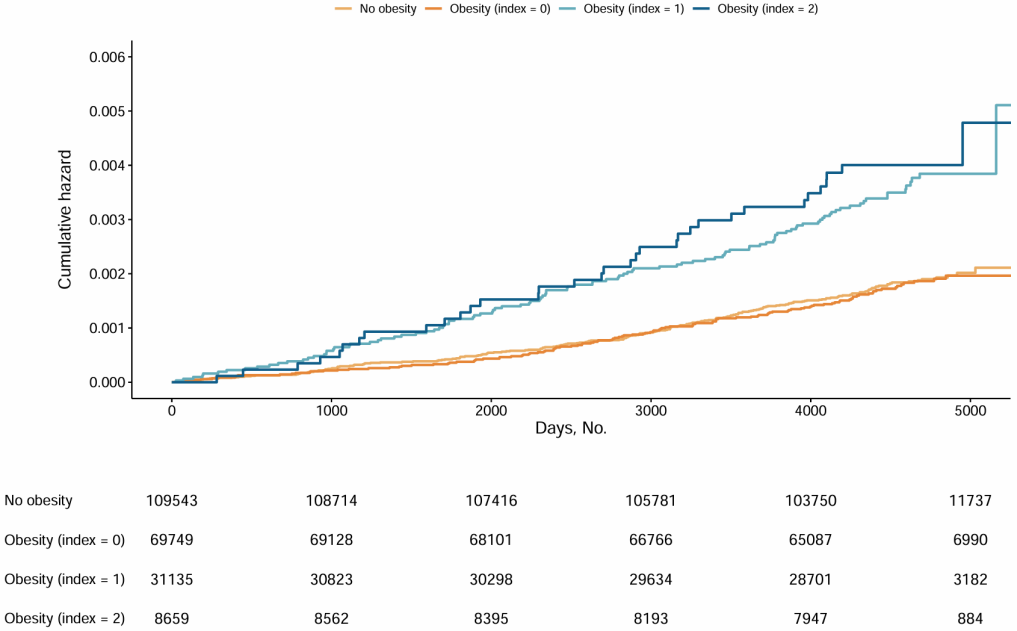


**C. Mood disorders**


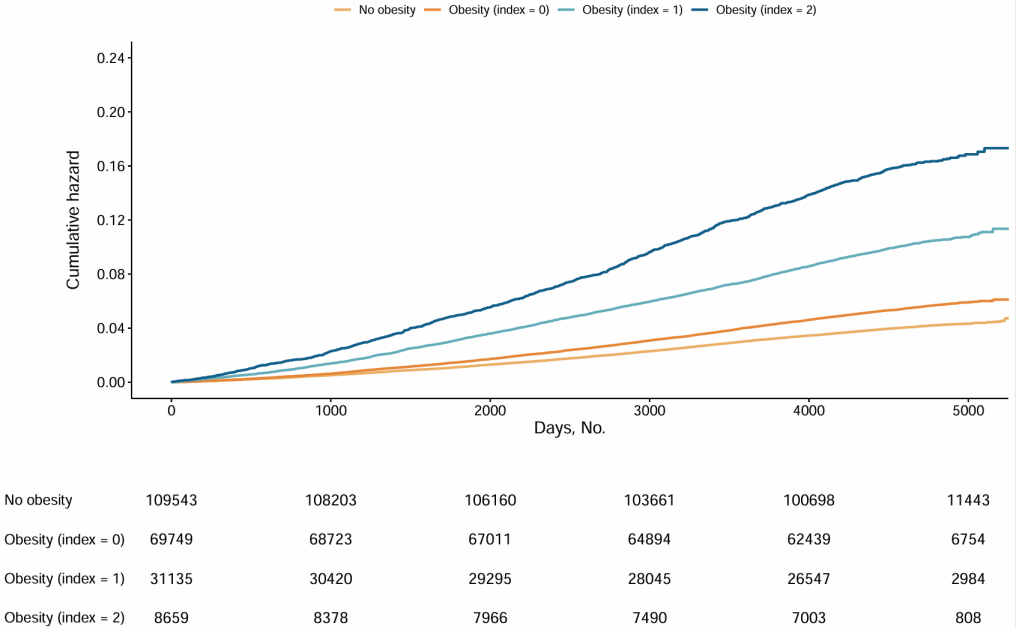


**D. Depression**


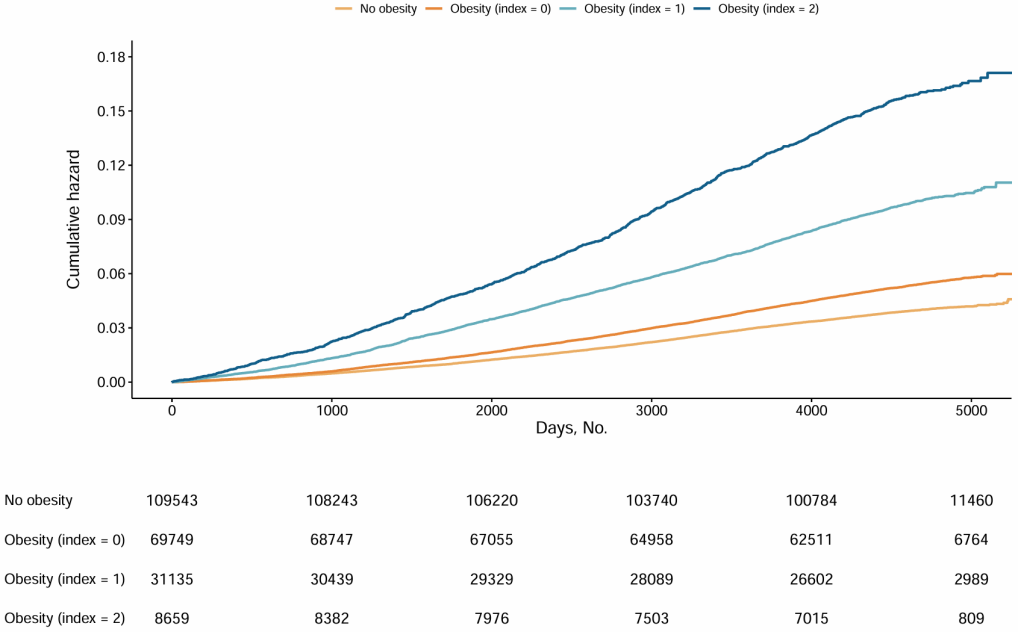


**E. Anxiety disorders**


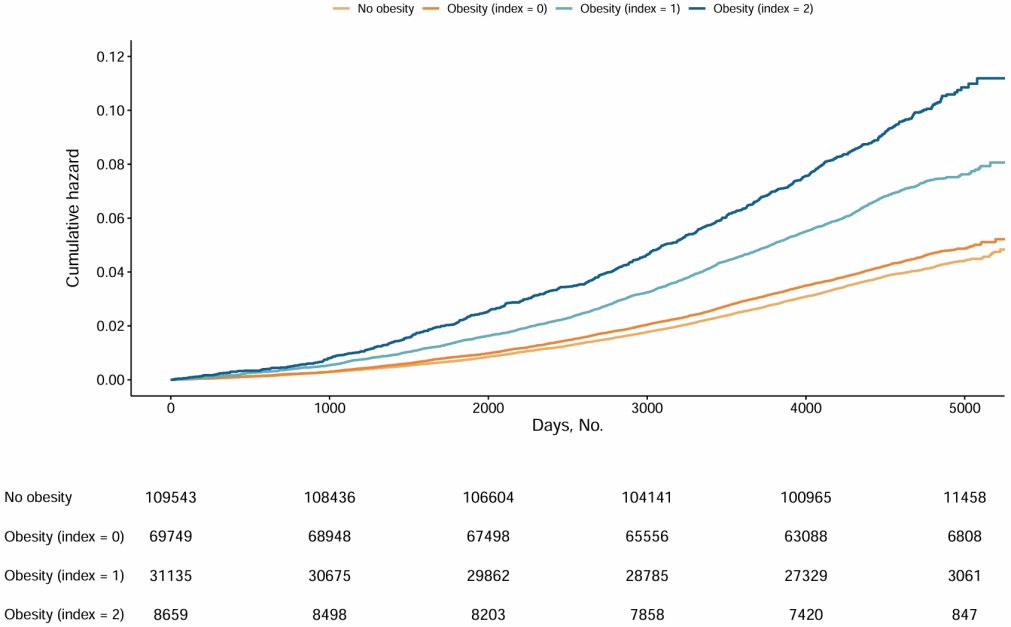


**F. PTSD**


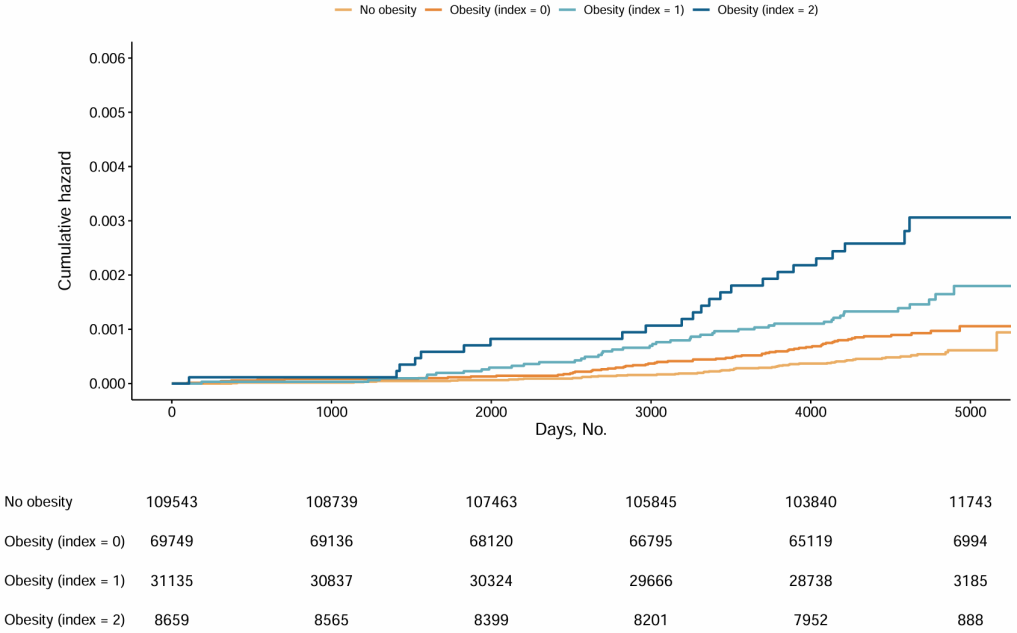


**G. Behavioral syndromes**


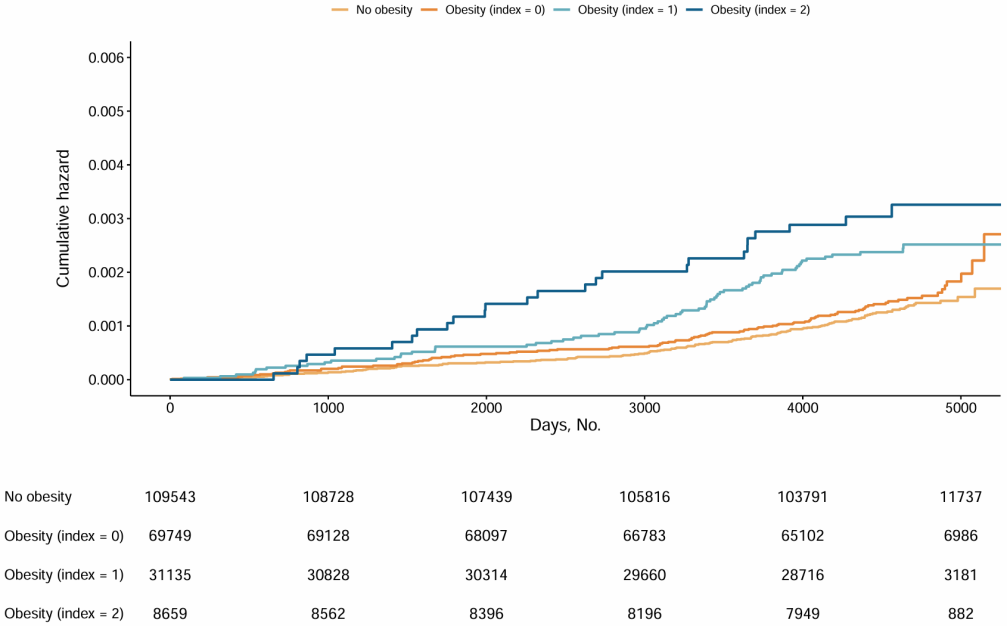


Figure S4. The cumulative hazard of subtypes of mental disorders corresponding to weight status and loneliness index.

(A) Substance use disorders; (B) Psychotic disorders; (C) Mood disorders; (D) Depression; (E) Anxiety disorders; (F) PTSD; (G) Behavioral syndromes.

Abbreviations: PTSD: post-traumatic stress disorder.

**A. Substance use disorders**


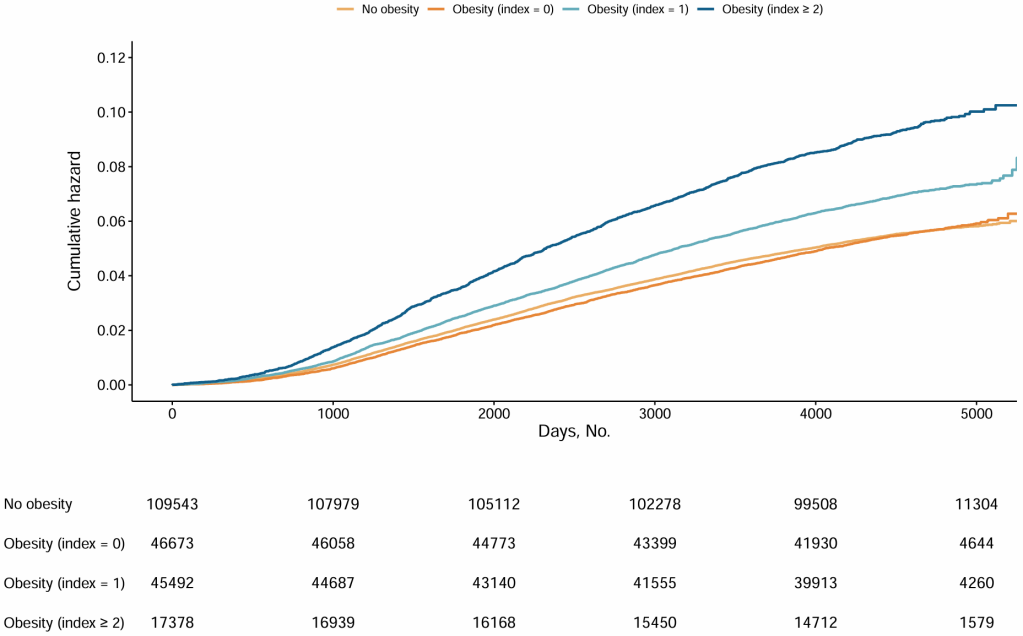


**B. Psychotic disorders**


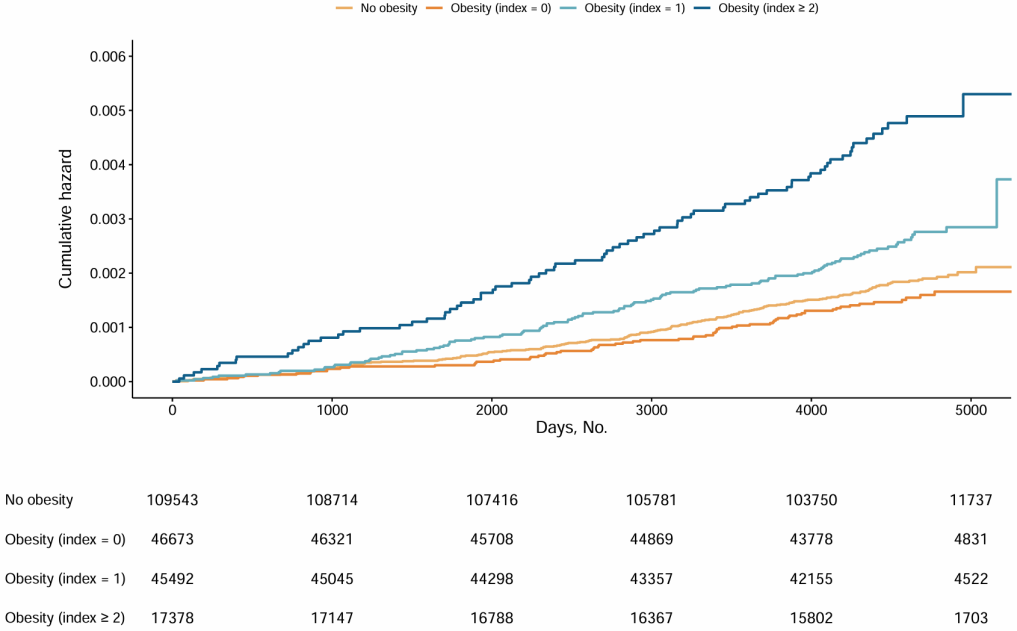


**C. Mood disorders**


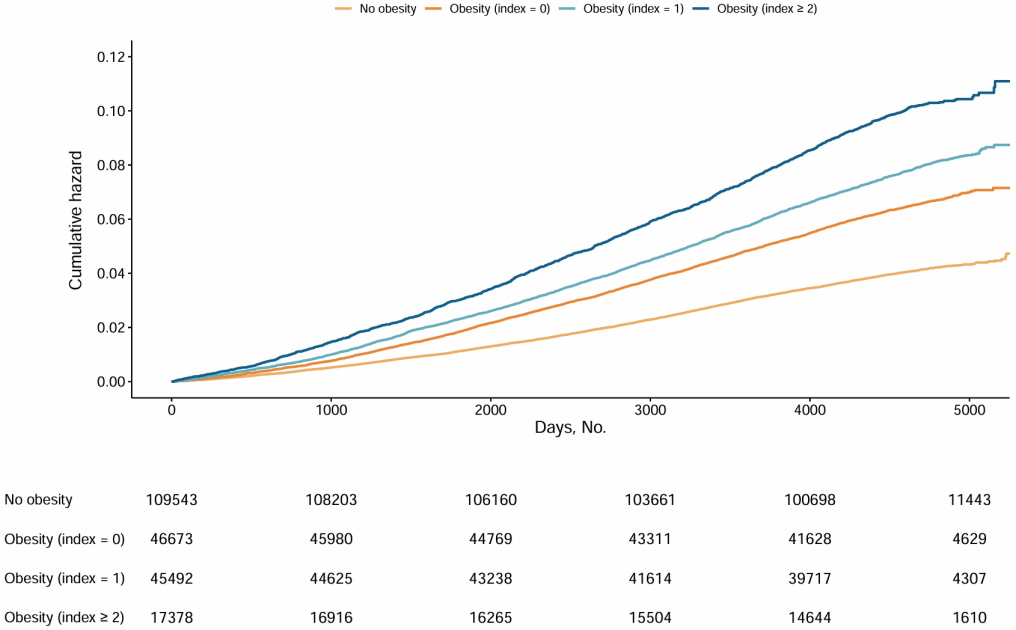


**D. Depression**


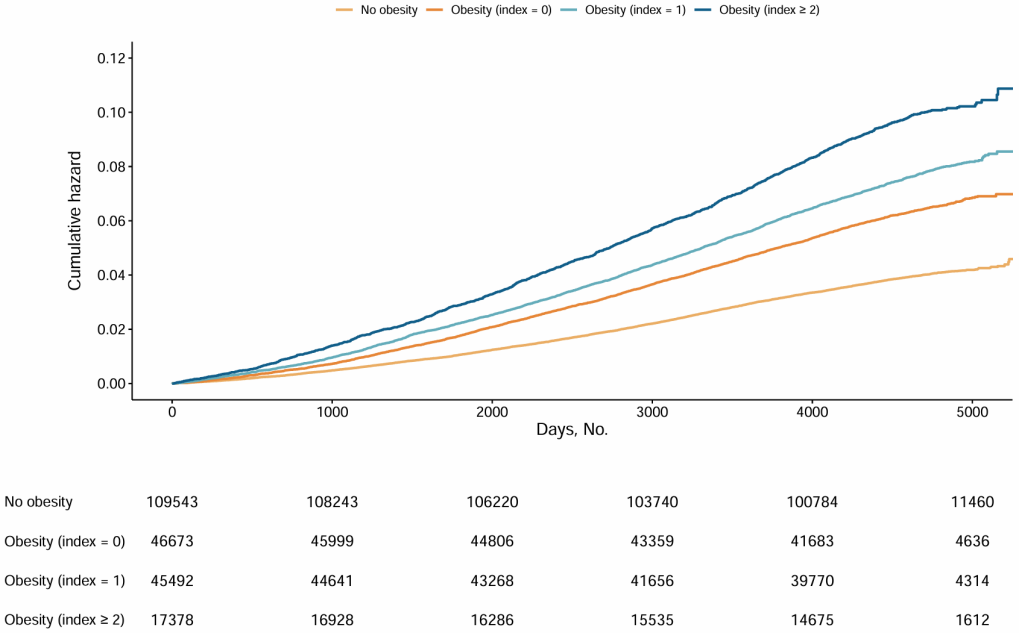


**E. Anxiety disorders**


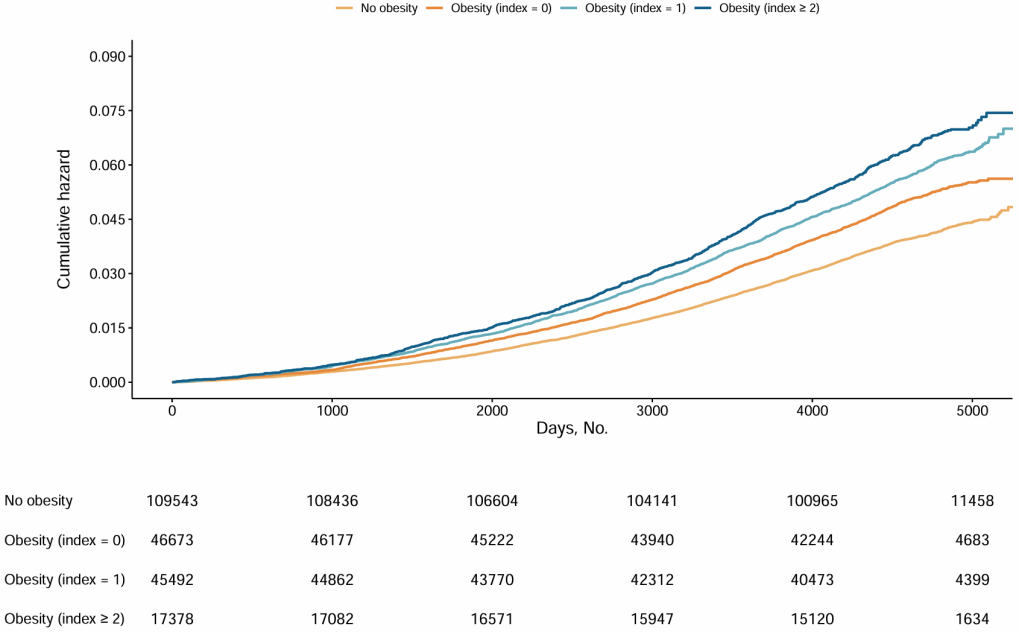


**F. PTSD**


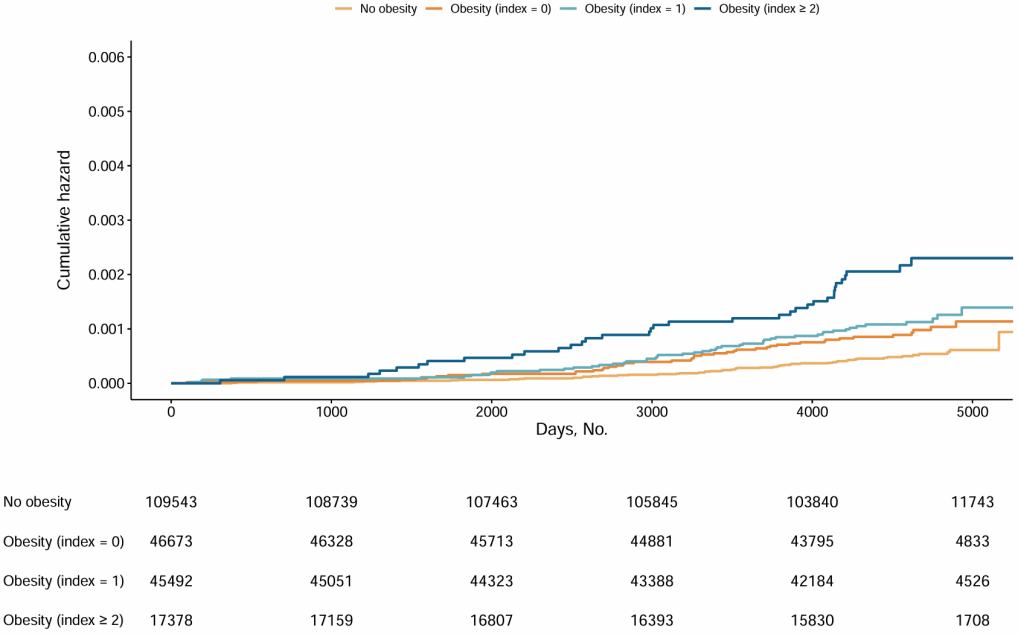


**G. Behavioral syndromes**


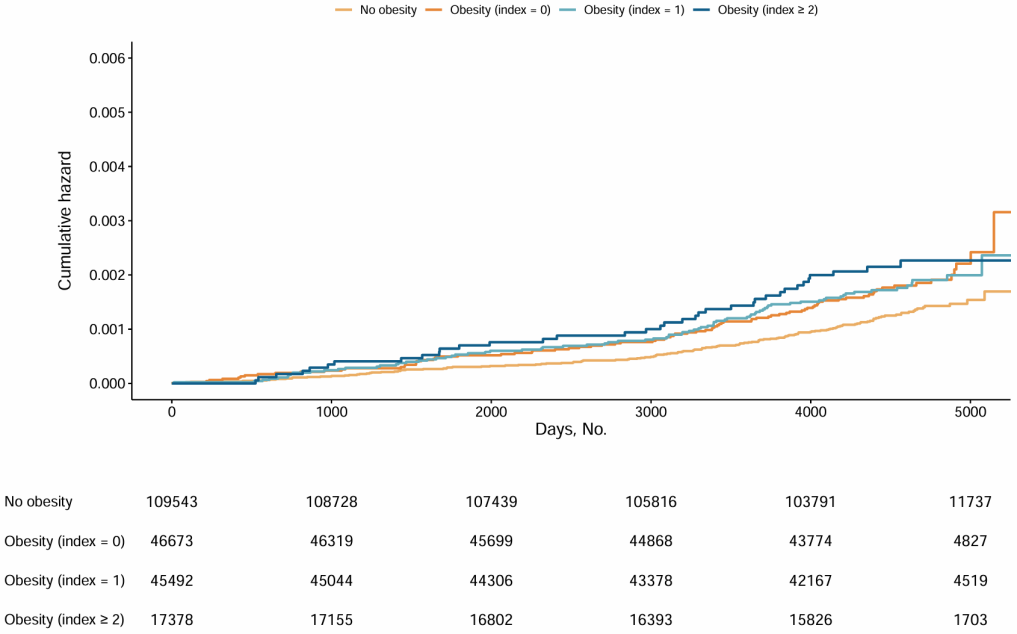


Figure S5. The cumulative hazard of subtypes of mental disorders corresponding to weight status and social isolation index.

(A) Substance use disorders; (B) Psychotic disorders; (C) Mood disorders; (D) Depression; (E) Anxiety disorders; (F) PTSD; (G) Behavioral syndromes.

Abbreviations: PTSD: post-traumatic stress disorder.

Table S1. Definition of loneliness and social isolation in the UK Biobank

| **Data field IDs** | **ACE touchscreen questions** | **Responses** | **Score** |
| --- | --- | --- | --- |
| **Loneliness** | | | |
| 2020 | Do you often feel lonely? | No | 0 |
|  |  | Yes | 1 |
| 2110 | How often are you able to confide in someone close to you? | Almost daily | 0 |
|  |  | 2-4 times a week | 0 |
|  |  | About once a week | 0 |
|  |  | About once a month | 0 |
|  |  | Once every few months | 1 |
|  |  | Never or almost never | 1 |
| **Social isolation** | | | |
| 709 | Including yourself, how many people are living together in your household? | Not living alone | 0 |
|  |  | Living alone | 1 |
| 1031 | How often do you visit friends or family or have them visit you? | Almost daily | 0 |
|  |  | 2-4 times a week | 0 |
|  |  | About once a week | 0 |
|  |  | About once a month | 1 |
|  |  | Once every few months | 1 |
|  |  | Never or almost never | 1 |
|  |  | No friends/family outside household | 1 |
| 6160 | Which of the following (sports club or gym, pub or social club, religious group, adult education class, other group activity) do you attend once a week or more often? | Sports club or gym | 0 |
|  |  | Pub or social club | 0 |
|  |  | Religious group | 0 |
|  |  | Adult education class | 0 |
|  |  | Other group activity | 0 |
|  |  | None of the above | 1 |

Table S2. Definition of mental disorders in the UK Biobank

| **Mental disorders** | **ICD 10** |
| --- | --- |
| All mental disorders | F00-F99 |
| Substance use disorders | F10-F19 |
| Psychotic disorders | F20-F29 |
| Mood disorders | F30-F39 |
| Depression | F32-F33 |
| Anxiety disorders | F40-F48 |
| Posttraumatic stress disorder (PTSD) | F43.1 |
| Behavioral syndromes | F50-F59 |

Table S3. Definitions of lifestyles in the UK Biobank

| **Lifestyles** | **Definition** |
| --- | --- |
| Diet | Healthy diet was defined as an adequate intake of at least 5 of the 10 dietary components (details were shown in Table S4) |
| Smoking status | The participants' current smoking status (current, previous, and never) was recorded, and no current smoking was on target. |
| Alcohol intake | Participants were asked about their drinking frequency and then further asked the amount of red wine (glasses), champagne plus white wine (glasses), beer plus cider (pints), spirits (measures), fortified wine (glasses), and other alcoholic drinks (glasses) they consumed on average in a week or a month. Alcohol intake in units per day was calculated by summing the average individual drinks per day according to alcohol unit reference. The units were then converted to grams assuming that one unit equals 8 g. No more than 14 g/day for women and 28 g/day for men was on target. |
| Physical activity | The number of days of moderate/vigorous physical activity in a typical week and duration of moderate/vigorous physical activity on a typical day was recorded. The number of days was multiplied by the time duration per day to calculate the weekly total amount of moderate or vigorous physical activity. Sufficient physical activity was defined as ≥150 minutes of moderate activity or ≥75 minutes of vigorous activity per week. |
| Sleep | Sleep duration was recorded by asking "About how many hours sleep do you get in every 24 hours? (please include naps)". Adequate sleep duration was defined as 7-8 hours/day. |

Detailed information on questionnaires can be obtained through the UK Biobank website (https://biobank.ndph.ox.ac.uk/showcase/).

Table S4. Components of diet quality score used in the UK Biobank

| **Components** | **Data field IDs** | **Amount per serving** | **Intake goal** |
| --- | --- | --- | --- |
| Fruit | 1309 (pieces fresh fruit/day)  1319 (pieces dried fruit/day) | 1309: 1 piece  1319: 5 pieces | ≥3 servings/day |
| Vegetable | 1289 (tablespoons cooked vegetables/day)  1299 (salad/raw vegetables/day) | 3 heaped tablespoons | ≥3 servings/day |
| Fish | 1329 (oily fish/week)  1339 (non-oily fish/week) | Once/week | ≥2 servings/week |
| Processed meats | 1349 (processed meat/week or daily)  3680 (age when last ate meat) | 1349: 1 piece/day  3680: 0 pieces/day if indicated having never eaten meat | ≤1 serving/week |
| Unprocessed red meats | 1369 (beef/week or day)  1379 (lamb or mutton/week or day)  1389 (pork/week or day)  3680 (age when last ate meat) | 1359~1389: once/week  3680: 0 pieces/day if indicated having never eaten meat | ≤2 serving/week |
| Whole grains | 1438, 1448 (whole meal/whole grain bread slices/week)  1458, 1468 (bran/oat/muesli cereal bowls/week) | 1438/1448: 1 slice/day  1458/1468: 1 bowl/day | ≥3 servings/day |
| Refined grains | 1438  1448 (white, brown, other bread slices/week)  1458  1468 (biscuit, other cereals/week) | 1438/1448: 1 slice/day  1458/1468: 1 bowl/day | ≤2 servings/day |
| Vegetable oils | 1428 (Flora Pro-Active/Benecol spread)  2654 (Flora Pro-Active/Benecol, soft margarine-, olive oil based-, polyunsaturated/sunflower oil based-, other low/reduced fat spread)  1438 (bread slices/week) | 1 serving/day if in combination with eating at least 2 slices of bread (ID 1438) | ≥2 servings/day |
| Dairy | 1408 (cheese/week)  1418 (milk type) | 1408: 1 piece/day  1418: 1 glass/day if consumption of any type of milk | ≥2 servings/day |
| Sugar-  sweetened beverages | 6144 (never consumes drinks containing sugar) | 0 servings | Don’t drink |

Table S5. Definitions of chronic diseases in the UK Biobank

| **Chronic diseases** | **Self-reported** | **ICD 9** | **ICD 10** |
| --- | --- | --- | --- |
| Cardiovascular Disease***** | 6150 (1, 2, 3), 20002 (1074, 1075, 1081, 1583, 1086, 1491) | 410-414, 430-434, 436 | I20-I25, I60-I64 |
| Hypertension | 6150 (4), 20002 (1065, 1072), 6153 (2), 6177 (2) | 401-405 | I10-I13,  I15, O10 |
| Cancer | 20001 | 140-208 | C00-C97 |
| Respiratory disease† | 20002 (1112, 1113, 1472), 6152 (6) | 491, 492, 496 | J40-J44, J47 |
| Type 2 diabetes | Participants with type 2 diabetes were identified according to the algorithm developed by the UK Biobank. This algorithm takes into account multiple aspects of information such as self-reported medical history and medication, and has been proven valid with 96% accuracy.^[1]^ | | |

***** Contains coronary heart disease and stroke.

† Contains chronic obstructive pulmonary disease and emphysema.

**References**

[1] Eastwood SV, Mathur R, Atkinson M, et al. (2016) Algorithms for the capture and adjudication of prevalent and incident diabetes in UK Biobank. PLOS ONE 11: e162388

Table S6. Baseline characteristics of non-obese participants

|  | **Overall (N = 149034)** | **Loneliness** | | | | **Social isolation** | | | |
| --- | --- | --- | --- | --- | --- | --- | --- | --- | --- |
|  |  | **Index = 0 (N = 105043)** | **Index = 1 (N = 36242)** | **Index = 2 (N = 7749)** | ***P* value** | **Index = 0 (N = 68180)** | **Index = 1 (N = 60100)** | **Index ≥ 2 (N = 20754)** | ***P* value** |
| Age, years | 49.0 [57.0, 62.0] | 56.0 [49.0, 62.0] | 57.0 [49.0, 63.0] | 56.0 [49.0, 62.0] | < 0.001 | 57.0 [49.0, 63.0] | 56.0 [49.0, 62.0] | 56.0 [49.0, 62.0] | < 0.001 |
| Female | 97873 (65.7%) | 70235 (66.9%) | 22999 (63.5%) | 4639 (59.9%) | < 0.001 | 46899 (68.8%) | 39213 (65.2%) | 11761 (56.7%) | < 0.001 |
| White British | 133276 (89.4%) | 94905 (90.3%) | 31728 (87.5%) | 6643 (85.7%) | < 0.001 | 61882 (90.8%) | 53600 (89.2%) | 17794 (85.7%) | < 0.001 |
| Townsend deprivation index | -3.8 [-2.4, 0.0] | -2.5 [-3.8, -0.2] | -2.1 [-3.6, 0.5] | -1.7 [-3.5, 1.4] | < 0.001 | -2.7 [-3.9, -0.7] | -2.2 [-3.7, 0.3] | -1.4 [-3.3, 1.6] | < 0.001 |
| College/university degree | 60455 (40.6%) | 45157 (43.0%) | 12870 (35.5%) | 2428 (31.3%) | < 0.001 | 27588 (40.5%) | 24318 (40.5%) | 8549 (41.2%) | 0.140 |
| Healthy diet | 30026 (20.1%) | 21513 (20.5%) | 7089 (19.6%) | 1424 (18.4%) | < 0.001 | 13610 (20.0%) | 12177 (20.3%) | 4239 (20.4%) | 0.230 |
| No current smoking | 133201 (89.4%) | 95568 (91.0%) | 31356 (86.5%) | 6277 (81.0%) | < 0.001 | 62852 (92.2%) | 53144 (88.4%) | 17205 (82.9%) | < 0.001 |
| Moderate alcohol consumption | 111049 (74.5%) | 78017 (74.3%) | 27181 (75.0%) | 5851 (75.5%) | 0.003 | 49185 (72.1%) | 45467 (75.7%) | 16397 (79.0%) | < 0.001 |
| Sufficient physical activity | 86869 (58.3%) | 61934 (59.0%) | 20702 (57.1%) | 4233 (54.6%) | < 0.001 | 42659 (62.6%) | 33906 (56.4%) | 10304 (49.6%) | < 0.001 |
| Healthy sleep duration | 107127 (71.9%) | 78355 (74.6%) | 24143 (66.6%) | 4629 (59.7%) | < 0.001 | 50664 (74.3%) | 42627 (70.9%) | 13836 (66.7%) | < 0.001 |
| The number of chronic diseases |  |  | | | < 0.001 |  | | | < 0.001 |
| 0 | 109310 (73.3%) | 77935 (74.2%) | 25972 (71.7%) | 5403 (69.7%) |  | 50469 (74.0%) | 44074 (73.3%) | 14767 (71.2%) |  |
| 1-2 | 38721 (26.0%) | 26507 (25.2%) | 9960 (27.5%) | 2254 (29.1%) |  | 17315 (25.4%) | 15621 (26.0%) | 5785 (27.9%) |  |
| ≥3 | 1003 (0.7%) | 601 (0.6%) | 310 (0.9%) | 92 (1.2%) |  | 396 (0.6%) | 405 (0.7%) | 202 (1.0%) |  |

Data are n (%) for categorical variables, mean (SD) for normally distributed continuous variables, and median [IQR] for nonnormally distributed continuous variables.

Table S7. Distribution of loneliness and social isolation in the obese and non-obese participants

|  | **Obesity (n = 109543)** | **No obesity (n = 149034)** | ***P*** value |
| --- | --- | --- | --- |
| Loneliness |  |  | < 0.001 |
| Index = 0 | 69749 (63.67%) | 105043 (70.48%) |  |
| Index = 1 | 31135 (28.42%) | 36242 (24.32%) |  |
| Index = 2 | 8659 (7.90%) | 7749 (5.20%) |  |
| Social isolation |  |  | < 0.001 |
| Index = 0 | 46673 (42.61%) | 68180 (45.75%) |  |
| Index = 1 | 45492 (41.53%) | 60100 (40.33%) |  |
| Index ≥ 2 | 17378 (15.86%) | 20754 (13.93%) |  |

Table S8. Associations of individual indicator of loneliness and social isolation with risk of mental disorders in the obese participants

|  | **Cases/**  **Person-Years** | **HR (95% CI)** | **PAF (%) (95% CI)** |
| --- | --- | --- | --- |
| **All mental disorders** |  |  |  |
| Items of loneliness |  |  |  |
| Often feel lonely |  |  | 11.7 (11.0, 12.5) |
| No | 12201/989510 | Ref. |  |
| Yes | 6079/257689 | 1.71 (1.66-1.76) |  |
| Able to confide |  |  | 2.9 (2.2, 3.6) |
| Once a month or more | 13492/972024 | Ref. |  |
| Less than once a month | 4788/275176 | 1.15 (1.11-1.19) |  |
| Items of social isolation |  |  |  |
| Live alone |  |  | 3.3 (2.6, 4.0) |
| No | 13658/1007967 | Ref. |  |
| Yes | 4622/239233 | 1.18 (1.14-1.23) |  |
| Contact with family/friends |  |  | 1.3 (0.6, 1.9) |
| Once a week or more | 14350/988593 | Ref. |  |
| Less than once a week | 3930/258607 | 1.07 (1.04-1.11) |  |
| Engaging in group activities once a week or more often |  |  | 2.4 (1.5, 3.3) |
| Yes | 11432/828787 | Ref. |  |
| No | 6848/418413 | 1.08 (1.04-1.11) |  |
| **Substance use disorders** |  |  |  |
| Items of loneliness |  |  |  |
| Often feel lonely |  |  | 6.2 (5.0, 7.4) |
| No | 4877/1021773 | Ref. |  |
| Yes | 2075/279298 | 1.32 (1.25-1.39) |  |
| Able to confide |  |  | 2.5 (1.3, 3.7) |
| Once a month or more | 5020/1012114 | Ref. |  |
| Less than once a month | 1932/288957 | 1.11 (1.06-1.18) |  |
| Items of social isolation |  |  |  |
| Live alone |  |  | 4.0 (2.8, 5.2) |
| No | 5026/1049011 | Ref. |  |
| Yes | 1926/252060 | 1.21 (1.15-1.28) |  |
| Contact with family/friends |  |  | 1.3 (0.2, 2.4) |
| Once a week or more | 5358/1030957 | Ref. |  |
| Less than once a week | 1594/270114 | 1.07 (1.01-1.13) |  |
| Engaging in group activities once a week or more often |  |  | 3.6 (2.0, 5.2) |
| Yes | 4276/862706 | Ref. |  |
| No | 2676/438365 | 1.12 (1.06-1.17) |  |
| **Psychotic disorders** |  |  |  |
| Items of loneliness |  |  |  |
| Often feel lonely |  |  | 22.7 (14.7, 30.7) |
| No | 153/1047646 | Ref. |  |
| Yes | 108/290663 | 2.18 (1.69-2.82) |  |
| Able to confide |  |  | 0.8 (-6.4, 8.0) |
| Once a month or more | 193/1039040 | Ref. |  |
| Less than once a month | 68/299269 | 1.03 (0.78-1.36) |  |
| Items of social isolation |  |  |  |
| Live alone |  |  | 31.7 (23.8, 39.6) |
| No | 138/1076078 | Ref. |  |
| Yes | 123/262232 | 2.98 (2.31-3.85) |  |
| Contact with family/friends |  |  | 6.1 (-1.0, 13.1) |
| Once a week or more | 191/1059792 | Ref. |  |
| Less than once a week | 70/278517 | 1.29 (0.97-1.70) |  |
| Engaging in group activities once a week or more often |  |  | -0.2 (-9.7, 9.4) |
| Yes | 167/885284 | Ref. |  |
| No | 94/453026 | 0.99 (0.76-1.28) |  |
| **Mood disorders** |  |  |  |
| Items of loneliness |  |  |  |
| Often feel lonely |  |  | 24.3 (22.9, 25.7) |
| No | 4372/1026650 | Ref. |  |
| Yes | 3306/272865 | 2.41 (2.30-2.53) |  |
| Able to confide |  |  | 4.9 (3.6, 6.1) |
| Once a month or more | 5652/1010391 | Ref. |  |
| Less than once a month | 2026/289124 | 1.23 (1.17-1.30) |  |
| Items of social isolation |  |  |  |
| Live alone |  |  | 4.6 (3.4, 5.9) |
| No | 5735/1046991 | Ref. |  |
| Yes | 1943/252524 | 1.23 (1.17-1.30) |  |
| Contact with family/friends |  |  | 2.1 (1.0, 3.3) |
| Once a week or more | 5998/1029411 | Ref. |  |
| Less than once a week | 1680/270104 | 1.11 (1.05-1.17) |  |
| Engaging in group activities once a week or more often |  |  | 3.1 (1.4, 4.8) |
| Yes | 4701/861576 | Ref. |  |
| No | 2977/437939 | 1.09 (1.04-1.14) |  |
| **Depression** |  |  |  |
| Items of loneliness |  |  |  |
| Often feel lonely |  |  | 24.3 (22.9, 25.7) |
| No | 4276/1027338 | Ref. |  |
| Yes | 3236/273314 | 2.41 (2.30-2.53) |  |
| Able to confide |  |  | 5.1 (3.9, 6.4) |
| Once a month or more | 5515/1011308 | Ref. |  |
| Less than once a month | 1997/289344 | 1.25 (1.18-1.31) |  |
| Items of social isolation |  |  |  |
| Live alone |  |  | 4.5 (3.3, 5.8) |
| No | 5619/1047757 | Ref. |  |
| Yes | 1893/252896 | 1.23 (1.16-1.30) |  |
| Contact with family/friends |  |  | 2.1 (1.0, 3.2) |
| Once a week or more | 5873/1030272 | Ref. |  |
| Less than once a week | 1639/270381 | 1.11 (1.05-1.17) |  |
| Engaging in group activities once a week or more often |  |  | 3.3 (1.6, 5.0) |
| Yes | 4588/862369 | Ref. |  |
| No | 2924/438283 | 1.09 (1.04-1.15) |  |
| **Anxiety disorders** |  |  |  |
| Items of loneliness |  |  |  |
| Often feel lonely |  |  | 17.4 (15.8, 19.0) |
| No | 3522/1033385 | Ref. |  |
| Yes | 2106/281816 | 1.91 (1.81-2.02) |  |
| Able to confide |  |  | 3.4 (2.0, 4.9) |
| Once a month or more | 4222/1021745 | Ref. |  |
| Less than once a month | 1406/293457 | 1.16 (1.09-1.23) |  |
| Items of social isolation |  |  |  |
| Live alone |  |  | 2.2 (0.8, 3.6) |
| No | 4314/1057911 | Ref. |  |
| Yes | 1314/257290 | 1.11 (1.04-1.18) |  |
| Contact with family/friends |  |  | 1.4 (0.1, 2.7) |
| Once a week or more | 4459/1041400 | Ref. |  |
| Less than once a week | 1169/273802 | 1.07 (1.01-1.15) |  |
| Engaging in group activities once a week or more often |  |  | 2.6 (0.6, 4.6) |
| Yes | 3509/870987 | Ref. |  |
| No | 2119/444215 | 1.07 (1.02-1.13) |  |
| **PTSD** |  |  |  |
| Items of loneliness |  |  |  |
| Often feel lonely |  |  | 24.0 (12.4, 35.5) |
| No | 73/1048018 | Ref. |  |
| Yes | 55/290937 | 2.24 (1.56-3.22) |  |
| Able to confide |  |  | 3.9 (-6.5, 14.4) |
| Once a month or more | 93/1039525 | Ref. |  |
| Less than once a month | 35/299430 | 1.16 (0.78-1.72) |  |
| Items of social isolation |  |  |  |
| Live alone |  |  | 7.3 (-2.6, 17.2) |
| No | 94/1076290 | Ref. |  |
| Yes | 34/262664 | 1.36 (0.91-2.05) |  |
| Contact with family/friends |  |  | 9.4 (-1.2, 19.9) |
| Once a week or more | 89/1060249 | Ref. |  |
| Less than once a week | 39/278706 | 1.44 (0.98-2.11) |  |
| Engaging in group activities once a week or more often |  |  | 3.9 (-10.1, 18.0) |
| Yes | 78/885673 | Ref. |  |
| No | 50/453282 | 1.11 (0.77-1.59) |  |
| **Behavioral syndromes** |  |  |  |
| Items of loneliness |  |  |  |
| Often feel lonely |  |  | 10.2 (2.7, 17.7) |
| No | 147/1047689 | Ref. |  |
| Yes | 54/290914 | 1.59 (1.15-2.19) |  |
| Able to confide |  |  | 12.2 (3.3, 21.1) |
| Once a month or more | 131/1039349 | Ref. |  |
| Less than once a month | 70/299253 | 1.53 (1.14-2.05) |  |
| Items of social isolation |  |  |  |
| Live alone |  |  | 0.2 (-6.9, 7.2) |
| No | 161/1075951 | Ref. |  |
| Yes | 40/262652 | 1.01 (0.70-1.44) |  |
| Contact with family/friends |  |  | 4.6 (-3.5, 12.7) |
| Once a week or more | 147/1060026 | Ref. |  |
| Less than once a week | 54/278576 | 1.20 (0.88-1.65) |  |
| Engaging in group activities once a week or more often |  |  | -1.3 (-10.9, 8.3) |
| Yes | 139/885403 | Ref. |  |
| No | 62/453199 | 0.95 (0.70-1.30) |  |

Abbreviations: CI, Confidence interval; HR, Hazard ratio; PTSD: post-traumatic stress disorder; PAF: population attributed fraction.

Adjusted for age, sex, ethnicity, Townsend deprivation index, education, diet, smoking status, alcohol consumption, exercise, sleep duration, and the number of chronic diseases.

Table S9. Associations of joint exposure of loneliness and social isolation with risk of mental disorders in the obese participants

|  | **Cases/Person-Years** | **HR (95% CI) (model 1)** | **HR (95% CI) (model 2)** | **HR (95% CI) (model 3)** |
| --- | --- | --- | --- | --- |
| **All mental disorders** |  |  |  |  |
| No loneliness & No isolated | 4388/390550 | Ref. | Ref. | Ref. |
| No loneliness & Isolated | 5328/416490 | 1.14 (1.10-1.19) | 1.11 (1.07-1.15) | 1.06 (1.02-1.10) |
| Loneliness & No isolated | 2508/149291 | 1.50 (1.43-1.58) | 1.46 (1.39-1.53) | 1.40 (1.33-1.47) |
| Loneliness & Isolated | 6056/290869 | 1.87 (1.80-1.95) | 1.72 (1.65-1.79) | 1.55 (1.49-1.61) |
| *P* value for trend |  | < 0.001 | < 0.001 | < 0.001 |
| **Substance use disorders** |  |  |  |  |
| No loneliness & No isolated | 1635/402875 | Ref. | Ref. | Ref. |
| No loneliness & Isolated | 2168/430502 | 1.24 (1.16-1.32) | 1.18 (1.10-1.26) | 1.11 (1.04-1.19) |
| Loneliness & No isolated | 825/157945 | 1.29 (1.18-1.40) | 1.22 (1.12-1.32) | 1.14 (1.04-1.24) |
| Loneliness & Isolated | 2324/309748 | 1.85 (1.74-1.97) | 1.59 (1.50-1.70) | 1.38 (1.30-1.48) |
| *P* value for trend |  | < 0.001 | < 0.001 | < 0.001 |
| **Psychotic disorders** |  |  |  |  |
| No loneliness & No isolated | 42/411582 | Ref. | Ref. | Ref. |
| No loneliness & Isolated | 77/442027 | 1.71 (1.17-2.49) | 1.59 (1.09-2.31) | 1.54 (1.05-2.24) |
| Loneliness & No isolated | 27/162349 | 1.63 (1.01-2.64) | 1.52 (0.94-2.47) | 1.47 (0.91-2.39) |
| Loneliness & Isolated | 115/322352 | 3.51 (2.46-4.99) | 2.87 (2.00-4.11) | 2.64 (1.84-3.79) |
| *P* value for trend |  | < 0.001 | < 0.001 | < 0.001 |
| **Mood disorders** |  |  |  |  |
| No loneliness & No isolated | 1620/403699 | Ref. | Ref. | Ref. |
| No loneliness & Isolated | 1953/432532 | 1.13 (1.05-1.20) | 1.08 (1.01-1.15) | 1.03 (0.97-1.10) |
| Loneliness & No isolated | 1213/155796 | 1.95 (1.81-2.10) | 1.88 (1.74-2.02) | 1.80 (1.67-1.94) |
| Loneliness & Isolated | 2892/307488 | 2.36 (2.22-2.51) | 2.16 (2.03-2.30) | 1.97 (1.85-2.09) |
| *P* value for trend |  | < 0.001 | < 0.001 | < 0.001 |
| **Depression** |  |  |  |  |
| No loneliness & No isolated | 1577/403998 | Ref. | Ref. | Ref. |
| No loneliness & Isolated | 1914/432815 | 1.13 (1.06-1.21) | 1.09 (1.02-1.16) | 1.04 (0.97-1.11) |
| Loneliness & No isolated | 1194/155949 | 1.97 (1.83-2.12) | 1.90 (1.76-2.04) | 1.82 (1.68-1.96) |
| Loneliness & Isolated | 2827/307890 | 2.37 (2.22-2.52) | 2.18 (2.04-2.32) | 1.98 (1.86-2.11) |
| *P* value for trend |  | < 0.001 | < 0.001 | < 0.001 |
| **Anxiety disorders** |  |  |  |  |
| No loneliness & No isolated | 1341/406010 | Ref. | Ref. | Ref. |
| No loneliness & Isolated | 1532/435896 | 1.07 (0.99-1.15) | 1.03 (0.96-1.11) | 1.01 (0.93-1.08) |
| Loneliness & No isolated | 845/158784 | 1.62 (1.48-1.76) | 1.57 (1.44-1.71) | 1.52 (1.39-1.66) |
| Loneliness & Isolated | 1910/314511 | 1.85 (1.73-1.99) | 1.74 (1.62-1.86) | 1.62 (1.51-1.74) |
| *P* value for trend |  | < 0.001 | < 0.001 | < 0.001 |
| **PTSD** |  |  |  |  |
| No loneliness & No isolated | 22/411676 | Ref. | Ref. | Ref. |
| No loneliness & Isolated | 39/442200 | 1.66 (0.98-2.80) | 1.55 (0.92-2.61) | 1.47 (0.87-2.49) |
| Loneliness & No isolated | 21/162373 | 2.42 (1.33-4.40) | 2.30 (1.26-4.18) | 2.14 (1.17-3.90) |
| Loneliness & Isolated | 46/322706 | 2.68 (1.61-4.46) | 2.38 (1.42-3.98) | 2.06 (1.22-3.46) |
| *P* value for trend |  | < 0.001 | < 0.001 | 0.007 |
| **Behavioral syndromes** |  |  |  |  |
| No loneliness & No isolated | 47/411582 | Ref. | Ref. | Ref. |
| No loneliness & Isolated | 56/442100 | 1.11 (0.75-1.64) | 1.15 (0.78-1.70) | 1.16 (0.78-1.71) |
| Loneliness & No isolated | 38/162288 | 2.05 (1.34-3.14) | 2.10 (1.37-3.22) | 2.07 (1.35-3.18) |
| Loneliness & Isolated | 60/322632 | 1.63 (1.12-2.39) | 1.65 (1.12-2.43) | 1.62 (1.10-2.41) |
| *P* value for trend |  | 0.011 | 0.011 | 0.014 |

Abbreviations: CI, Confidence interval; HR, Hazard ratio; PTSD: post-traumatic stress disorder.

Adjusted for age, sex, ethnicity, Townsend deprivation index, education, diet, smoking status, alcohol consumption, exercise, sleep duration, and the number of chronic diseases.

Table S10. The standardized mean differences of age, sex, and assessment center before and after propensity score matching

|  | **Before PSM** | **After PSM** |
| --- | --- | --- |
| Age | 0.151 | 0.031 |
| Sex | 0.267 | 0.014 |
| Assessment center | 0.213 | 0.117 |

Table S11. Subgroup analyses of the associations between loneliness and the risk of mental disorders in the obese participants stratified by sociodemographic characteristics

|  | **Cases/N** | **Loneliness, HR (95% CI)** | | | ***P* value for interaction** |
| --- | --- | --- | --- | --- | --- |
|  |  | **Index = 0** | **Index = 1** | **Index = 2** |  |
| **All mental disorders** |  |  |  |  |  |
| Age, years |  |  |  |  | < 0.001 |
| < 60 | 9433/60987 | Ref. | 1.46 (1.39-1.52) | 1.88 (1.77-2.00) |  |
| ≥ 60 | 8847/48556 | Ref. | 1.31 (1.25-1.37) | 1.60 (1.49-1.72) |  |
| Sex |  |  |  |  | 0.162 |
| Female | 9925/57676 | Ref. | 1.42 (1.36-1.48) | 1.78 (1.67-1.89) |  |
| Male | 8355/51867 | Ref. | 1.34 (1.28-1.41) | 1.74 (1.62-1.86) |  |
| Ethnicity |  |  |  |  | 0.085 |
| White British | 16386/97698 | Ref. | 1.40 (1.35-1.45) | 1.75 (1.66-1.84) |  |
| Others | 1894/11845 | Ref. | 1.27 (1.15-1.40) | 1.79 (1.57-2.05) |  |
| Townsend deprivation index |  |  |  |  | 0.978 |
| Low | 7689/54766 | Ref. | 1.39 (1.32-1.46) | 1.76 (1.63-1.90) |  |
| High | 10591/54777 | Ref. | 1.38 (1.32-1.44) | 1.75 (1.65-1.85) |  |
| Education |  |  |  |  | 0.095 |
| College/university degree | 3577/27854 | Ref. | 1.39 (1.29-1.49) | 1.95 (1.75-2.17) |  |
| Others | 14703/81689 | Ref. | 1.38 (1.33-1.43) | 1.72 (1.63-1.81) |  |
| **Substance use disorders** |  |  |  |  |  |
| Age, years |  |  |  |  | 0.701 |
| < 60 | 3903/60987 | Ref. | 1.20 (1.12-1.28) | 1.41 (1.28-1.55) |  |
| ≥ 60 | 3049/48556 | Ref. | 1.20 (1.11-1.29) | 1.38 (1.22-1.56) |  |
| Sex |  |  |  |  | 0.230 |
| Female | 2863/57676 | Ref. | 1.22 (1.12-1.32) | 1.33 (1.18-1.49) |  |
| Male | 4089/51867 | Ref. | 1.18 (1.10-1.26) | 1.45 (1.31-1.60) |  |
| Ethnicity |  |  |  |  | 0.157 |
| White British | 6173/97698 | Ref. | 1.22 (1.15-1.29) | 1.41 (1.30-1.53) |  |
| Others | 779/11845 | Ref. | 1.05 (0.90-1.23) | 1.30 (1.05-1.61) |  |
| Townsend deprivation index |  |  |  |  | 0.422 |
| Low | 2542/54766 | Ref. | 1.16 (1.06-1.26) | 1.36 (1.18-1.58) |  |
| High | 4410/54777 | Ref. | 1.22 (1.14-1.30) | 1.42 (1.30-1.55) |  |
| Education |  |  |  |  | 0.697 |
| College/university degree | 1211/27854 | Ref. | 1.20 (1.06-1.37) | 1.50 (1.24-1.82) |  |
| Others | 5741/81689 | Ref. | 1.19 (1.13-1.26) | 1.38 (1.27-1.50) |  |
| **Psychotic disorders** |  |  |  |  |  |
| Age, years |  |  |  |  | 0.299 |
| < 60 | 133/60987 | Ref. | 2.07 (1.43-3.00) | 1.64 (0.94-2.85) |  |
| ≥ 60 | 128/48556 | Ref. | 1.55 (1.06-2.26) | 2.15 (1.25-3.70) |  |
| Sex |  |  |  |  | 0.001 |
| Female | 123/57676 | Ref. | 1.12 (0.76-1.66) | 1.21 (0.66-2.20) |  |
| Male | 138/51867 | Ref. | 2.80 (1.94-4.06) | 2.79 (1.66-4.70) |  |
| Ethnicity |  |  |  |  | 0.834 |
| White British | 215/97698 | Ref. | 1.78 (1.34-2.38) | 1.74 (1.13-2.70) |  |
| Others | 46/11845 | Ref. | 1.95 (1.02-3.72) | 2.42 (1.03-5.66) |  |
| Townsend deprivation index |  |  |  |  | 0.061 |
| Low | 83/54766 | Ref. | 1.75 (1.11-2.73) | 0.69 (0.21-2.22) |  |
| High | 178/54777 | Ref. | 1.86 (1.34-2.57) | 2.25 (1.46-3.45) |  |
| Education |  |  |  |  | 0.039 |
| College/university degree | 50/27854 | Ref. | 2.01 (1.14-3.54) | 0.33 (0.04-2.43) |  |
| Others | 211/81689 | Ref. | 1.77 (1.32-2.38) | 2.18 (1.45-3.26) |  |
| **Mood disorders** |  |  |  |  |  |
| Age, years |  |  |  |  | 0.002 |
| < 60 | 4610/60987 | Ref. | 1.83 (1.71-1.95) | 2.61 (2.40-2.84) |  |
| ≥ 60 | 3068/48556 | Ref. | 1.57 (1.46-1.70) | 2.33 (2.09-2.60) |  |
| Sex |  |  |  |  | 0.060 |
| Female | 5042/57676 | Ref. | 1.69 (1.59-1.80) | 2.38 (2.19-2.58) |  |
| Male | 2636/51867 | Ref. | 1.78 (1.64-1.94) | 2.77 (2.48-3.09) |  |
| Ethnicity |  |  |  |  | 0.247 |
| White British | 6899/97698 | Ref. | 1.74 (1.65-1.83) | 2.48 (2.31-2.66) |  |
| Others | 779/11845 | Ref. | 1.59 (1.35-1.86) | 2.67 (2.20-3.25) |  |
| Townsend deprivation index |  |  |  |  | 0.359 |
| Low | 3240/54766 | Ref. | 1.65 (1.53-1.78) | 2.40 (2.15-2.67) |  |
| High | 4438/54777 | Ref. | 1.77 (1.66-1.89) | 2.58 (2.37-2.80) |  |
| Education |  |  |  |  | 0.758 |
| College/university degree | 1606/27854 | Ref. | 1.72 (1.55-1.92) | 2.60 (2.24-3.02) |  |
| Others | 6072/81689 | Ref. | 1.72 (1.63-1.82) | 2.48 (2.30-2.67) |  |
| **Depression** |  |  |  |  |  |
| Age, years |  |  |  |  | 0.004 |
| < 60 | 4508/60987 | Ref. | 1.82 (1.70-1.94) | 2.64 (2.42-2.86) |  |
| ≥ 60 | 3004/48556 | Ref. | 1.58 (1.46-1.71) | 2.35 (2.11-2.63) |  |
| Sex |  |  |  |  | 0.056 |
| Female | 4948/57676 | Ref. | 1.69 (1.59-1.79) | 2.40 (2.21-2.61) |  |
| Male | 2564/51867 | Ref. | 1.78 (1.63-1.94) | 2.80 (2.51-3.14) |  |
| Ethnicity |  |  |  |  | 0.217 |
| White British | 6762/97698 | Ref. | 1.73 (1.64-1.82) | 2.49 (2.32-2.67) |  |
| Others | 750/11845 | Ref. | 1.61 (1.37-1.90) | 2.80 (2.30-3.42) |  |
| Townsend deprivation index |  |  |  |  | 0.248 |
| Low | 3178/54766 | Ref. | 1.64 (1.52-1.77) | 2.41 (2.16-2.69) |  |
| High | 4334/54777 | Ref. | 1.78 (1.67-1.90) | 2.61 (2.40-2.84) |  |
| Education |  |  |  |  | 0.656 |
| College/university degree | 1551/27854 | Ref. | 1.71 (1.54-1.92) | 2.67 (2.30-3.11) |  |
| Others | 5961/81689 | Ref. | 1.72 (1.62-1.82) | 2.50 (2.32-2.69) |  |
| **Anxiety disorders** |  |  |  |  |  |
| Age, years |  |  |  |  | 0.001 |
| < 60 | 3119/60987 | Ref. | 1.61 (1.49-1.74) | 2.10 (1.89-2.33) |  |
| ≥ 60 | 2509/48556 | Ref. | 1.36 (1.25-1.49) | 1.75 (1.53-1.99) |  |
| Sex |  |  |  |  | 0.966 |
| Female | 3785/57676 | Ref. | 1.48 (1.38-1.59) | 1.94 (1.76-2.14) |  |
| Male | 1843/51867 | Ref. | 1.51 (1.37-1.67) | 1.98 (1.72-2.28) |  |
| Ethnicity |  |  |  |  | 0.627 |
| White British | 5073/97698 | Ref. | 1.49 (1.40-1.58) | 1.93 (1.77-2.10) |  |
| Others | 555/11845 | Ref. | 1.53 (1.27-1.84) | 2.16 (1.70-2.75) |  |
| Townsend deprivation index |  |  |  |  | 0.377 |
| Low | 2517/54766 | Ref. | 1.53 (1.40-1.66) | 1.86 (1.63-2.13) |  |
| High | 3111/54777 | Ref. | 1.47 (1.36-1.59) | 1.99 (1.80-2.21) |  |
| Education |  |  |  |  | 0.294 |
| College/university degree | 1154/27854 | Ref. | 1.51 (1.33-1.72) | 2.24 (1.86-2.69) |  |
| Others | 4474/81689 | Ref. | 1.49 (1.39-1.59) | 1.89 (1.73-2.07) |  |
| **PTSD** |  |  |  |  |  |
| Age, years |  |  |  |  | 0.030 |
| < 60 | 99/60987 | Ref. | 1.72 (1.09-2.69) | 3.00 (1.76-5.09) |  |
| ≥ 60 | 29/48556 | Ref. | 0.94 (0.41-2.15) | 0.50 (0.07-3.72) |  |
| Sex |  |  |  |  | 0.644 |
| Female | 59/57676 | Ref. | 1.26 (0.71-2.23) | 1.71 (0.80-3.65) |  |
| Male | 69/51867 | Ref. | 1.67 (0.98-2.86) | 3.13 (1.64-5.97) |  |
| Ethnicity |  |  |  |  | 0.696 |
| White British | 111/97698 | Ref. | 1.57 (1.04-2.38) | 2.38 (1.40-4.06) |  |
| Others | 17/11845 | Ref. | 0.95 (0.31-2.94) | 2.20 (0.64-7.52) |  |
| Townsend deprivation index |  |  |  |  | 0.221 |
| Low | 52/54766 | Ref. | 2.17 (1.21-3.91) | 2.72 (1.17-6.37) |  |
| High | 76/54777 | Ref. | 1.10 (0.65-1.86) | 2.16 (1.19-3.93) |  |
| Education |  |  |  |  | 0.488 |
| College/university degree | 38/27854 | Ref. | 1.36 (0.64-2.87) | 3.60 (1.55-8.35) |  |
| Others | 90/81689 | Ref. | 1.50 (0.95-2.38) | 2.03 (1.12-3.71) |  |
| **Behavioral syndromes** |  |  |  |  |  |
| Age, years |  |  |  |  | 0.960 |
| < 60 | 93/60987 | Ref. | 1.59 (1.01-2.49) | 1.97 (1.07-3.62) |  |
| ≥ 60 | 108/48556 | Ref. | 1.51 (1.00-2.28) | 2.01 (1.07-3.77) |  |
| Sex |  |  |  |  | 0.734 |
| Female | 28/57676 | Ref. | 1.36 (0.59-3.17) | 2.47 (0.87-7.01) |  |
| Male | 173/51867 | Ref. | 1.59 (1.15-2.20) | 1.91 (1.18-3.10) |  |
| Ethnicity |  |  |  |  | 0.950 |
| White British | 174/97698 | Ref. | 1.59 (1.14-2.20) | 2.03 (1.26-3.26) |  |
| Others | 27/11845 | Ref. | 1.35 (0.59-3.11) | 1.81 (0.58-5.62) |  |
| Townsend deprivation index |  |  |  |  | 0.153 |
| Low | 88/54766 | Ref. | 1.12 (0.69-1.80) | 1.44 (0.65-3.16) |  |
| High | 113/54777 | Ref. | 2.02 (1.35-3.04) | 2.54 (1.48-4.36) |  |
| Education |  |  |  |  | 0.482 |
| College/university degree | 51/27854 | Ref. | 1.17 (0.62-2.21) | 2.28 (0.99-5.24) |  |
| Others | 150/81689 | Ref. | 1.71 (1.21-2.42) | 1.95 (1.17-3.26) |  |

Abbreviations: CI, Confidence interval; HR, Hazard ratio; PTSD: post-traumatic stress disorder.

Adjusted for age, sex, ethnicity, Townsend deprivation index, education, diet, smoking status, alcohol consumption, exercise, sleep duration, and the number of chronic diseases.

Table S12. Subgroup analyses of the associations between social isolation and the risk of mental disorders in the obese participants stratified by sociodemographic characteristics

|  | **Cases/N** | **Social isolation, HR (95% CI)** | | | ***P* value for interaction** |
| --- | --- | --- | --- | --- | --- |
|  |  | **Index = 0** | **Index = 1** | **Index ≥ 2** |  |
| **All mental disorders** |  |  |  |  |  |
| Age, years |  |  |  |  | 0.525 |
| < 60 | 9433/60987 | Ref. | 1.08 (1.03-1.13) | 1.24 (1.17-1.31) |  |
| ≥ 60 | 8847/48556 | Ref. | 1.15 (1.09-1.20) | 1.29 (1.22-1.38) |  |
| Sex |  |  |  |  | 0.415 |
| Female | 9925/57676 | Ref. | 1.09 (1.04-1.14) | 1.21 (1.14-1.28) |  |
| Male | 8355/51867 | Ref. | 1.13 (1.08-1.19) | 1.34 (1.26-1.42) |  |
| Ethnicity |  |  |  |  | 0.764 |
| White British | 16386/97698 | Ref. | 1.11 (1.07-1.15) | 1.26 (1.20-1.31) |  |
| Others | 1894/11845 | Ref. | 1.08 (0.97-1.20) | 1.27 (1.13-1.44) |  |
| Townsend deprivation index |  |  |  |  | 0.001 |
| Low | 7689/54766 | Ref. | 1.05 (1.00-1.10) | 1.16 (1.09-1.25) |  |
| High | 10591/54777 | Ref. | 1.16 (1.11-1.21) | 1.33 (1.26-1.40) |  |
| Education |  |  |  |  | 0.089 |
| College/university degree | 3577/27854 | Ref. | 1.06 (0.98-1.14) | 1.32 (1.20-1.44) |  |
| Others | 14703/81689 | Ref. | 1.12 (1.08-1.16) | 1.25 (1.19-1.31) |  |
| **Substance use disorders** |  |  |  |  |  |
| Age, years |  |  |  |  | 0.430 |
| < 60 | 3903/60987 | Ref. | 1.11 (1.03-1.19) | 1.25 (1.15-1.37) |  |
| ≥ 60 | 3049/48556 | Ref. | 1.23 (1.13-1.33) | 1.45 (1.31-1.60) |  |
| Sex |  |  |  |  | 0.178 |
| Female | 2863/57676 | Ref. | 1.11 (1.02-1.21) | 1.27 (1.15-1.41) |  |
| Male | 4089/51867 | Ref. | 1.18 (1.10-1.27) | 1.36 (1.25-1.48) |  |
| Ethnicity |  |  |  |  | 0.538 |
| White British | 6173/97698 | Ref. | 1.17 (1.10-1.24) | 1.34 (1.25-1.44) |  |
| Others | 779/11845 | Ref. | 1.04 (0.88-1.23) | 1.27 (1.05-1.53) |  |
| Townsend deprivation index |  |  |  |  | 0.066 |
| Low | 2542/54766 | Ref. | 1.10 (1.01-1.19) | 1.26 (1.12-1.42) |  |
| High | 4410/54777 | Ref. | 1.21 (1.12-1.29) | 1.38 (1.28-1.50) |  |
| Education |  |  |  |  | 0.372 |
| College/university degree | 1211/27854 | Ref. | 1.10 (0.97-1.25) | 1.42 (1.21-1.65) |  |
| Others | 5741/81689 | Ref. | 1.17 (1.10-1.24) | 1.32 (1.22-1.42) |  |
| **Psychotic disorders** |  |  |  |  |  |
| Age, years |  |  |  |  | 0.189 |
| < 60 | 133/60987 | Ref. | 1.18 (0.78-1.81) | 2.22 (1.42-3.46) |  |
| ≥ 60 | 128/48556 | Ref. | 2.10 (1.36-3.24) | 2.97 (1.81-4.90) |  |
| Sex |  |  |  |  | 0.003 |
| Female | 123/57676 | Ref. | 1.24 (0.83-1.86) | 1.35 (0.81-2.25) |  |
| Male | 138/51867 | Ref. | 2.06 (1.31-3.25) | 4.39 (2.73-7.03) |  |
| Ethnicity |  |  |  |  | 0.057 |
| White British | 215/97698 | Ref. | 1.34 (0.97-1.85) | 2.26 (1.58-3.23) |  |
| Others | 46/11845 | Ref. | 4.42 (1.69-11.58) | 5.91 (2.14-16.33) |  |
| Townsend deprivation index |  |  |  |  | 0.030 |
| Low | 83/54766 | Ref. | 1.01 (0.63-1.63) | 1.57 (0.85-2.88) |  |
| High | 178/54777 | Ref. | 2.16 (1.43-3.25) | 3.44 (2.23-5.30) |  |
| Education |  |  |  |  | 0.767 |
| College/university degree | 50/27854 | Ref. | 1.65 (0.84-3.21) | 2.19 (1.01-4.74) |  |
| Others | 211/81689 | Ref. | 1.55 (1.10-2.17) | 2.61 (1.80-3.77) |  |
| **Mood disorders** |  |  |  |  |  |
| Age, years |  |  |  |  | 0.525 |
| < 60 | 4610/60987 | Ref. | 1.07 (1.00-1.15) | 1.32 (1.21-1.42) |  |
| ≥ 60 | 3068/48556 | Ref. | 1.15 (1.06-1.25) | 1.35 (1.22-1.49) |  |
| Sex |  |  |  |  | 0.001 |
| Female | 5042/57676 | Ref. | 1.07 (1.00-1.13) | 1.22 (1.13-1.32) |  |
| Male | 2636/51867 | Ref. | 1.17 (1.07-1.28) | 1.53 (1.37-1.70) |  |
| Ethnicity |  |  |  |  | 0.752 |
| White British | 6899/97698 | Ref. | 1.10 (1.04-1.16) | 1.31 (1.23-1.40) |  |
| Others | 779/11845 | Ref. | 1.14 (0.96-1.34) | 1.42 (1.17-1.71) |  |
| Townsend deprivation index |  |  |  |  | 0.049 |
| Low | 3240/54766 | Ref. | 1.06 (0.98-1.14) | 1.22 (1.09-1.35) |  |
| High | 4438/54777 | Ref. | 1.14 (1.07-1.23) | 1.41 (1.30-1.52) |  |
| Education |  |  |  |  | 0.252 |
| College/university degree | 1606/27854 | Ref. | 1.05 (0.94-1.17) | 1.38 (1.21-1.58) |  |
| Others | 6072/81689 | Ref. | 1.12 (1.05-1.18) | 1.31 (1.22-1.41) |  |
| **Depression** |  |  |  |  |  |
| Age, years |  |  |  |  | 0.545 |
| < 60 | 4508/60987 | Ref. | 1.07 (1.01-1.15) | 1.32 (1.22-1.43) |  |
| ≥ 60 | 3004/48556 | Ref. | 1.15 (1.06-1.25) | 1.35 (1.21-1.49) |  |
| Sex |  |  |  |  | 0.001 |
| Female | 4948/57676 | Ref. | 1.07 (1.01-1.14) | 1.22 (1.13-1.33) |  |
| Male | 2564/51867 | Ref. | 1.16 (1.06-1.27) | 1.52 (1.37-1.69) |  |
| Ethnicity |  |  |  |  | 0.417 |
| White British | 6762/97698 | Ref. | 1.09 (1.04-1.16) | 1.31 (1.22-1.40) |  |
| Others | 750/11845 | Ref. | 1.17 (0.99-1.39) | 1.50 (1.24-1.82) |  |
| Townsend deprivation index |  |  |  |  | 0.037 |
| Low | 3178/54766 | Ref. | 1.05 (0.97-1.13) | 1.22 (1.10-1.35) |  |
| High | 4334/54777 | Ref. | 1.15 (1.08-1.24) | 1.41 (1.30-1.53) |  |
| Education |  |  |  |  | 0.147 |
| College/university degree | 1551/27854 | Ref. | 1.04 (0.93-1.17) | 1.41 (1.24-1.62) |  |
| Others | 5961/81689 | Ref. | 1.12 (1.06-1.18) | 1.31 (1.21-1.40) |  |
| **Anxiety disorders** |  |  |  |  |  |
| Age, years |  |  |  |  | 0.684 |
| < 60 | 3119/60987 | Ref. | 1.08 (1.00-1.17) | 1.18 (1.06-1.30) |  |
| ≥ 60 | 2509/48556 | Ref. | 1.08 (0.99-1.18) | 1.18 (1.05-1.32) |  |
| Sex |  |  |  |  | 0.024 |
| Female | 3785/57676 | Ref. | 1.04 (0.97-1.12) | 1.08 (0.99-1.19) |  |
| Male | 1843/51867 | Ref. | 1.16 (1.05-1.29) | 1.37 (1.21-1.56) |  |
| Ethnicity |  |  |  |  | 0.325 |
| White British | 5073/97698 | Ref. | 1.09 (1.03-1.16) | 1.16 (1.07-1.26) |  |
| Others | 555/11845 | Ref. | 0.99 (0.81-1.20) | 1.24 (0.99-1.54) |  |
| Townsend deprivation index |  |  |  |  | 0.207 |
| Low | 2517/54766 | Ref. | 1.06 (0.97-1.15) | 1.10 (0.98-1.25) |  |
| High | 3111/54777 | Ref. | 1.11 (1.02-1.20) | 1.22 (1.11-1.35) |  |
| Education |  |  |  |  | 0.125 |
| College/university degree | 1154/27854 | Ref. | 1.01 (0.89-1.15) | 1.28 (1.09-1.50) |  |
| Others | 4474/81689 | Ref. | 1.10 (1.03-1.17) | 1.15 (1.05-1.25) |  |
| **PTSD** |  |  |  |  |  |
| Age, years |  |  |  |  | 0.905 |
| < 60 | 99/60987 | Ref. | 1.04 (0.65-1.65) | 1.56 (0.93-2.63) |  |
| ≥ 60 | 29/48556 | Ref. | 1.33 (0.56-3.16) | 2.89 (1.11-7.50) |  |
| Sex |  |  |  |  | 0.634 |
| Female | 59/57676 | Ref. | 0.91 (0.51-1.65) | 1.33 (0.67-2.63) |  |
| Male | 69/51867 | Ref. | 1.29 (0.73-2.30) | 2.28 (1.22-4.26) |  |
| Ethnicity |  |  |  |  | 0.635 |
| White British | 111/97698 | Ref. | 1.08 (0.70-1.66) | 1.62 (0.98-2.66) |  |
| Others | 17/11845 | Ref. | 1.27 (0.36-4.52) | 3.05 (0.88-10.60) |  |
| Townsend deprivation index |  |  |  |  | 0.992 |
| Low | 52/54766 | Ref. | 1.08 (0.58-1.99) | 1.72 (0.82-3.62) |  |
| High | 76/54777 | Ref. | 1.11 (0.64-1.93) | 1.83 (1.02-3.30) |  |
| Education |  |  |  |  | 0.669 |
| College/university degree | 38/27854 | Ref. | 1.37 (0.63-3.00) | 2.52 (1.09-5.81) |  |
| Others | 90/81689 | Ref. | 1.00 (0.61-1.62) | 1.54 (0.89-2.67) |  |
| **Behavioral syndromes** |  |  |  |  |  |
| Age, years |  |  |  |  | 0.823 |
| < 60 | 93/60987 | Ref. | 1.07 (0.68-1.69) | 1.18 (0.66-2.09) |  |
| ≥ 60 | 108/48556 | Ref. | 0.97 (0.64-1.47) | 1.09 (0.62-1.91) |  |
| Sex |  |  |  |  | 0.856 |
| Female | 28/57676 | Ref. | 0.90 (0.39-2.10) | 1.25 (0.45-3.46) |  |
| Male | 173/51867 | Ref. | 1.03 (0.74-1.43) | 1.12 (0.73-1.73) |  |
| Ethnicity |  |  |  |  | 0.235 |
| White British | 174/97698 | Ref. | 0.91 (0.65-1.27) | 1.12 (0.73-1.72) |  |
| Others | 27/11845 | Ref. | 1.98 (0.80-4.89) | 1.34 (0.42-4.33) |  |
| Townsend deprivation index |  |  |  |  | 0.805 |
| Low | 88/54766 | Ref. | 0.88 (0.56-1.40) | 1.09 (0.57-2.08) |  |
| High | 113/54777 | Ref. | 1.12 (0.73-1.70) | 1.21 (0.72-2.02) |  |
| Education |  |  |  |  | 0.034 |
| College/university degree | 51/27854 | Ref. | 1.88 (0.97-3.63) | 2.06 (0.94-4.53) |  |
| Others | 150/81689 | Ref. | 0.84 (0.59-1.19) | 0.93 (0.58-1.50) |  |

Abbreviations: CI, Confidence interval; HR, Hazard ratio; PTSD: post-traumatic stress disorder.

Adjusted for age, sex, ethnicity, Townsend deprivation index, education, diet, smoking status, alcohol consumption, exercise, sleep duration, and the number of chronic diseases.

Table S13. Associations of loneliness and social isolation with risk of mental disorders in the obese participants after excluding patients who developed mental disorders within two years from baseline

|  | **Cases/Person-Years** | **HR (95% CI) (model 1)** | **HR (95% CI) (model 2)** | **HR (95% CI) (model 3)** |
| --- | --- | --- | --- | --- |
| **All mental disorders** |  |  |  |  |
| Loneliness |  |  |  |  |
| Index = 0 | 9040/806251 | Ref. | Ref. | Ref. |
| Index = 1 | 5723/346884 | 1.48 (1.44-1.53) | 1.41 (1.37-1.46) | 1.36 (1.31-1.40) |
| Index = 2 | 2078/92473 | 2.04 (1.95-2.14) | 1.91 (1.82-2.00) | 1.71 (1.62-1.79) |
| *P* value for trend |  | < 0.001 | < 0.001 | < 0.001 |
| Social isolation |  |  |  |  |
| Index = 0 | 6395/539273 | Ref. | Ref. | Ref. |
| Index = 1 | 7191/515365 | 1.18 (1.14-1.22) | 1.14 (1.10-1.18) | 1.10 (1.06-1.14) |
| Index ≥ 2 | 3255/190969 | 1.45 (1.39-1.52) | 1.35 (1.29-1.41) | 1.22 (1.17-1.27) |
| *P* value for trend |  | < 0.001 | < 0.001 | < 0.001 |
| **Substance use disorders** |  |  |  |  |
| Loneliness |  |  |  |  |
| Index = 0 | 3506/828463 | Ref. | Ref. | Ref. |
| Index = 1 | 2088/362815 | 1.36 (1.29-1.44) | 1.26 (1.19-1.33) | 1.18 (1.12-1.25) |
| Index = 2 | 764/98829 | 1.83 (1.69-1.98) | 1.60 (1.48-1.73) | 1.36 (1.25-1.47) |
| *P* value for trend |  | < 0.001 | < 0.001 | < 0.001 |
| Social isolation |  |  |  |  |
| Index = 0 | 2270/556761 | Ref. | Ref. | Ref. |
| Index = 1 | 2724/534232 | 1.25 (1.18-1.32) | 1.19 (1.12-1.25) | 1.13 (1.07-1.20) |
| Index ≥ 2 | 1364/199114 | 1.68 (1.58-1.80) | 1.46 (1.37-1.57) | 1.27 (1.18-1.36) |
| *P* value for trend |  | < 0.001 | < 0.001 | < 0.001 |
| **Psychotic disorders** |  |  |  |  |
| Loneliness |  |  |  |  |
| Index = 0 | 101/846280 | Ref. | Ref. | Ref. |
| Index = 1 | 88/373575 | 1.98 (1.49-2.63) | 1.77 (1.33-2.37) | 1.71 (1.28-2.29) |
| Index = 2 | 28/102714 | 2.29 (1.51-3.48) | 1.93 (1.26-2.95) | 1.78 (1.16-2.72) |
| *P* value for trend |  | < 0.001 | < 0.001 | < 0.001 |
| Social isolation |  |  |  |  |
| Index = 0 | 61/568359 | Ref. | Ref. | Ref. |
| Index = 1 | 91/548129 | 1.55 (1.12-2.14) | 1.44 (1.04-1.99) | 1.41 (1.02-1.96) |
| Index ≥ 2 | 65/206081 | 2.96 (2.08-4.19) | 2.46 (1.73-3.52) | 2.36 (1.64-3.38) |
| *P* value for trend |  | < 0.001 | < 0.001 | < 0.001 |
| **Mood disorders** |  |  |  |  |
| Loneliness |  |  |  |  |
| Index = 0 | 3236/831992 | Ref. | Ref. | Ref. |
| Index = 1 | 2539/361836 | 1.81 (1.72-1.91) | 1.75 (1.66-1.84) | 1.68 (1.59-1.77) |
| Index = 2 | 1072/97599 | 2.86 (2.67-3.06) | 2.67 (2.49-2.87) | 2.42 (2.26-2.60) |
| *P* value for trend |  | < 0.001 | < 0.001 | < 0.001 |
| Social isolation |  |  |  |  |
| Index = 0 | 2558/556542 | Ref. | Ref. | Ref. |
| Index = 1 | 2922/534916 | 1.19 (1.13-1.26) | 1.13 (1.07-1.20) | 1.09 (1.03-1.15) |
| Index ≥ 2 | 1367/199969 | 1.50 (1.40-1.60) | 1.39 (1.31-1.49) | 1.28 (1.20-1.37) |
| *P* value for trend |  | < 0.001 | < 0.001 | < 0.001 |
| **Depression** |  |  |  |  |
| Loneliness |  |  |  |  |
| Index = 0 | 3168/832397 | Ref. | Ref. | Ref. |
| Index = 1 | 2479/362166 | 1.81 (1.72-1.91) | 1.74 (1.65-1.84) | 1.68 (1.59-1.77) |
| Index = 2 | 1060/97688 | 2.88 (2.69-3.09) | 2.70 (2.52-2.90) | 2.45 (2.28-2.63) |
| *P* value for trend |  | < 0.001 | < 0.001 | < 0.001 |
| Social isolation |  |  |  |  |
| Index = 0 | 2505/556872 | Ref. | Ref. | Ref. |
| Index = 1 | 2863/535225 | 1.19 (1.13-1.26) | 1.13 (1.08-1.20) | 1.09 (1.03-1.15) |
| Index ≥ 2 | 1339/200154 | 1.50 (1.40-1.60) | 1.40 (1.31-1.49) | 1.28 (1.20-1.37) |
| *P* value for trend |  | < 0.001 | < 0.001 | < 0.001 |
| **Anxiety disorders** |  |  |  |  |
| Loneliness |  |  |  |  |
| Index = 0 | 2648/836284 | Ref. | Ref. | Ref. |
| Index = 1 | 1794/366833 | 1.55 (1.46-1.65) | 1.50 (1.41-1.60) | 1.46 (1.37-1.55) |
| Index = 2 | 676/100029 | 2.15 (1.98-2.34) | 2.05 (1.88-2.23) | 1.91 (1.76-2.08) |
| *P* value for trend |  | < 0.001 | < 0.001 | < 0.001 |
| Social isolation |  |  |  |  |
| Index = 0 | 2001/560639 | Ref. | Ref. | Ref. |
| Index = 1 | 2197/539809 | 1.15 (1.08-1.22) | 1.10 (1.03-1.17) | 1.07 (1.01-1.14) |
| Index ≥ 2 | 920/202698 | 1.28 (1.19-1.39) | 1.22 (1.13-1.32) | 1.16 (1.07-1.25) |
| *P* value for trend |  | < 0.001 | < 0.001 | < 0.001 |
| **PTSD** |  |  |  |  |
| Loneliness |  |  |  |  |
| Index = 0 | 51/846488 | Ref. | Ref. | Ref. |
| Index = 1 | 39/373792 | 1.73 (1.14-2.63) | 1.68 (1.10-2.55) | 1.56 (1.02-2.37) |
| Index = 2 | 20/102741 | 3.24 (1.93-5.43) | 2.91 (1.73-4.91) | 2.42 (1.42-4.11) |
| *P* value for trend |  | < 0.001 | < 0.001 | < 0.001 |
| Social isolation |  |  |  |  |
| Index = 0 | 38/568446 | Ref. | Ref. | Ref. |
| Index = 1 | 43/548321 | 1.18 (0.76-1.82) | 1.13 (0.73-1.75) | 1.06 (0.68-1.65) |
| Index ≥ 2 | 29/206254 | 2.13 (1.31-3.45) | 1.94 (1.19-3.16) | 1.70 (1.03-2.79) |
| *P* value for trend |  | 0.003 | 0.009 | 0.038 |
| **Behavioral syndromes** |  |  |  |  |
| Loneliness |  |  |  |  |
| Index = 0 | 94/846305 | Ref. | Ref. | Ref. |
| Index = 1 | 63/373730 | 1.52 (1.10-2.09) | 1.51 (1.10-2.09) | 1.50 (1.09-2.07) |
| Index = 2 | 24/102696 | 2.11 (1.34-3.30) | 2.14 (1.36-3.36) | 2.05 (1.30-3.23) |
| *P* value for trend |  | < 0.001 | < 0.001 | < 0.001 |
| Social isolation |  |  |  |  |
| Index = 0 | 75/568339 | Ref. | Ref. | Ref. |
| Index = 1 | 73/548185 | 1.01 (0.73-1.40) | 1.04 (0.75-1.44) | 1.05 (0.76-1.46) |
| Index ≥ 2 | 33/206207 | 1.22 (0.81-1.84) | 1.18 (0.78-1.78) | 1.20 (0.79-1.84) |
| *P* value for trend |  | 0.373 | 0.454 | 0.397 |

Abbreviations: CI, Confidence interval; HR, Hazard ratio; PTSD: post-traumatic stress disorder.

Model 1: without adjustment.

Model 2: adjusted for age, sex, ethnicity, Townsend deprivation index, and education.

Model 3: adjusted for age, sex, ethnicity, Townsend deprivation index, education, diet, smoking status, alcohol consumption, exercise, sleep duration, and the number of chronic diseases.

Table S14. Associations of loneliness and social isolation with risk of mental disorders in the obese participants considering competing risk event

|  | **Cases/Person-Years** | **HR (95% CI) (model 1)** | **HR (95% CI) (model 2)** | **HR (95% CI) (model 3)** |
| --- | --- | --- | --- | --- |
| **All mental disorders** |  |  |  |  |
| Loneliness |  |  |  |  |
| Index = 0 | 9716/807040 | Ref. | Ref. | Ref. |
| Index = 1 | 6261/347454 | 1.50 (1.45-1.55) | 1.43 (1.38-1.47) | 1.37 (1.32-1.41) |
| Index = 2 | 2303/92705 | 2.08 (1.99-2.18) | 1.93 (1.85-2.02) | 1.73 (1.65-1.81) |
| *P* value for trend |  | < 0.001 | < 0.001 | < 0.001 |
| Social isolation |  |  |  |  |
| Index = 0 | 6896/539841 | Ref. | Ref. | Ref. |
| Index = 1 | 7804/516031 | 1.18 (1.14-1.22) | 1.14 (1.10-1.17) | 1.09 (1.05-1.13) |
| Index ≥ 2 | 3580/191327 | 1.46 (1.40-1.52) | 1.35 (1.29-1.40) | 1.20 (1.15-1.26) |
| *P* value for trend |  | < 0.001 | < 0.001 | < 0.001 |
| **Substance use disorders** |  |  |  |  |
| Loneliness |  |  |  |  |
| Index = 0 | 3803/833377 | Ref. | Ref. | Ref. |
| Index = 1 | 2291/367132 | 1.36 (1.29-1.44) | 1.26 (1.19-1.32) | 1.18 (1.12-1.24) |
| Index = 2 | 858/100562 | 1.86 (1.73-2.01) | 1.62 (1.50-1.74) | 1.37 (1.27-1.48) |
| *P* value for trend |  | < 0.001 | < 0.001 | < 0.001 |
| Social isolation |  |  |  |  |
| Index = 0 | 2460/560820 | Ref. | Ref. | Ref. |
| Index = 1 | 2984/538749 | 1.26 (1.19-1.32) | 1.19 (1.13-1.25) | 1.13 (1.07-1.19) |
| Index ≥ 2 | 1508/201501 | 1.69 (1.58-1.80) | 1.46 (1.36-1.55) | 1.25 (1.16-1.34) |
| *P* value for trend |  | < 0.001 | < 0.001 | < 0.001 |
| **Psychotic disorders** |  |  |  |  |
| Loneliness |  |  |  |  |
| Index = 0 | 119/853609 | Ref. | Ref. | Ref. |
| Index = 1 | 108/379469 | 2.03 (1.57-2.64) | 1.81 (1.39-2.35) | 1.74 (1.34-2.27) |
| Index = 2 | 34/105232 | 2.30 (1.57-3.37) | 1.92 (1.32-2.81) | 1.75 (1.19-2.58) |
| *P* value for trend |  | < 0.001 | < 0.001 | < 0.001 |
| Social isolation |  |  |  |  |
| Index = 0 | 69/573931 | Ref. | Ref. | Ref. |
| Index = 1 | 114/554832 | 1.70 (1.26-2.29) | 1.56 (1.16-2.11) | 1.52 (1.12-2.05) |
| Index ≥ 2 | 78/209547 | 3.06 (2.21-4.22) | 2.50 (1.81-3.46) | 2.33 (1.68-3.22) |
| *P* value for trend |  | < 0.001 | < 0.001 | < 0.001 |
| **Mood disorders** |  |  |  |  |
| Loneliness |  |  |  |  |
| Index = 0 | 3573/836231 | Ref. | Ref. | Ref. |
| Index = 1 | 2878/364578 | 1.84 (1.76-1.94) | 1.78 (1.69-1.87) | 1.71 (1.62-1.79) |
| Index = 2 | 1227/98706 | 2.91 (2.73-3.11) | 2.71 (2.54-2.90) | 2.46 (2.30-2.63) |
| *P* value for trend |  | < 0.001 | < 0.001 | < 0.001 |
| Social isolation |  |  |  |  |
| Index = 0 | 2833/559495 | Ref. | Ref. | Ref. |
| Index = 1 | 3275/538395 | 1.20 (1.14-1.26) | 1.13 (1.08-1.19) | 1.09 (1.04-1.15) |
| Index ≥ 2 | 1570/201625 | 1.52 (1.43-1.62) | 1.41 (1.33-1.50) | 1.29 (1.21-1.38) |
| *P* value for trend |  | < 0.001 | < 0.001 | < 0.001 |
| **Depression** |  |  |  |  |
| Loneliness |  |  |  |  |
| Index = 0 | 3491/836813 | Ref. | Ref. | Ref. |
| Index = 1 | 2809/365020 | 1.84 (1.76-1.94) | 1.78 (1.69-1.87) | 1.70 (1.61-1.79) |
| Index = 2 | 1212/98819 | 2.94 (2.77-3.14) | 2.74 (2.57-2.92) | 2.49 (2.33-2.65) |
| *P* value for trend |  | < 0.001 | < 0.001 | < 0.001 |
| Social isolation |  |  |  |  |
| Index = 0 | 2771/559947 | Ref. | Ref. | Ref. |
| Index = 1 | 3205/538818 | 1.20 (1.14-1.26) | 1.14 (1.08-1.19) | 1.09 (1.04-1.15) |
| Index ≥ 2 | 1536/201887 | 1.52 (1.42-1.62) | 1.41 (1.33-1.50) | 1.29 (1.21-1.38) |
| *P* value for trend |  | < 0.001 | < 0.001 | < 0.001 |
| **Anxiety disorders** |  |  |  |  |
| Loneliness |  |  |  |  |
| Index = 0 | 2873/841906 | Ref. | Ref. | Ref. |
| Index = 1 | 1998/371318 | 1.57 (1.49-1.67) | 1.52 (1.44-1.61) | 1.48 (1.40-1.57) |
| Index = 2 | 757/101978 | 2.17 (2.00-2.35) | 2.06 (1.90-2.24) | 1.92 (1.77-2.08) |
| *P* value for trend |  | < 0.001 | < 0.001 | < 0.001 |
| Social isolation |  |  |  |  |
| Index = 0 | 2186/564794 | Ref. | Ref. | Ref. |
| Index = 1 | 2421/544873 | 1.14 (1.08-1.21) | 1.10 (1.03-1.16) | 1.07 (1.01-1.13) |
| Index ≥ 2 | 1021/205534 | 1.27 (1.18-1.37) | 1.21 (1.12-1.30) | 1.14 (1.05-1.23) |
| *P* value for trend |  | < 0.001 | < 0.001 | < 0.001 |
| **PTSD** |  |  |  |  |
| Loneliness |  |  |  |  |
| Index = 0 | 61/853875 | Ref. | Ref. | Ref. |
| Index = 1 | 44/379793 | 1.61 (1.09-2.38) | 1.54 (1.04-2.29) | 1.44 (0.97-2.14) |
| Index = 2 | 23/105287 | 3.03 (1.88-4.90) | 2.69 (1.66-4.35) | 2.28 (1.40-3.72) |
| *P* value for trend |  | < 0.001 | < 0.001 | < 0.001 |
| Social isolation |  |  |  |  |
| Index = 0 | 43/574049 | Ref. | Ref. | Ref. |
| Index = 1 | 50/555109 | 1.20 (0.80-1.80) | 1.13 (0.75-1.70) | 1.08 (0.71-1.62) |
| Index ≥ 2 | 35/209797 | 2.20 (1.41-3.44) | 1.93 (1.23-3.05) | 1.73 (1.08-2.79) |
| *P* value for trend |  | < 0.001 | 0.006 | 0.028 |
| **Behavioral syndromes** |  |  |  |  |
| Loneliness |  |  |  |  |
| Index = 0 | 103/853682 | Ref. | Ref. | Ref. |
| Index = 1 | 72/379674 | 1.56 (1.16-2.11) | 1.55 (1.15-2.10) | 1.54 (1.14-2.09) |
| Index = 2 | 26/105246 | 2.03 (1.32-3.12) | 2.05 (1.33-3.16) | 1.98 (1.29-3.04) |
| *P* value for trend |  | < 0.001 | < 0.001 | < 0.001 |
| Social isolation |  |  |  |  |
| Index = 0 | 85/573870 | Ref. | Ref. | Ref. |
| Index = 1 | 80/554966 | 0.97 (0.71-1.31) | 0.99 (0.73-1.35) | 0.99 (0.73-1.35) |
| Index ≥ 2 | 36/209766 | 1.14 (0.77-1.69) | 1.08 (0.73-1.60) | 1.09 (0.74-1.61) |
| *P* value for trend |  | 0.570 | 0.710 | 0.690 |

Abbreviations: CI, Confidence interval; HR, Hazard ratio; PTSD: post-traumatic stress disorder.

Model 1: without adjustment.

Model 2: adjusted for age, sex, ethnicity, Townsend deprivation index, and education.

Model 3: adjusted for age, sex, ethnicity, Townsend deprivation index, education, diet, smoking status, alcohol consumption, exercise, sleep duration, and the number of chronic diseases.

Table S15. Associations of loneliness and social isolation with risk of mental disorders in the obese participants with the imputation of exposure data

|  | **Cases/Person-Years** | **HR (95% CI) (model 1)** | **HR (95% CI) (model 2)** | **HR (95% CI) (model 3)** |
| --- | --- | --- | --- | --- |
| **All mental disorders** |  |  |  |  |
| Loneliness |  |  |  |  |
| Index = 0 | 10900/874706 | Ref. | Ref. | Ref. |
| Index = 1 | 6736/370727 | 1.47 (1.42-1.51) | 1.40 (1.36-1.44) | 1.34 (1.30-1.38) |
| Index = 2 | 2489/100816 | 2.01 (1.92-2.10) | 1.87 (1.79-1.96) | 1.68 (1.61-1.76) |
| *P* value for trend |  | < 0.001 | < 0.001 | < 0.001 |
| Social isolation |  |  |  |  |
| Index = 0 | 7626/581165 | Ref. | Ref. | Ref. |
| Index = 1 | 8603/559372 | 1.18 (1.14-1.21) | 1.13 (1.10-1.17) | 1.09 (1.05-1.12) |
| Index ≥ 2 | 3896/205712 | 1.46 (1.40-1.51) | 1.35 (1.30-1.40) | 1.21 (1.17-1.26) |
| *P* value for trend |  | < 0.001 | < 0.001 | < 0.001 |
| **Substance use disorders** |  |  |  |  |
| Loneliness |  |  |  |  |
| Index = 0 | 4278/904393 | Ref. | Ref. | Ref. |
| Index = 1 | 2469/391776 | 1.33 (1.27-1.40) | 1.23 (1.17-1.30) | 1.16 (1.11-1.22) |
| Index = 2 | 929/109296 | 1.80 (1.68-1.93) | 1.57 (1.46-1.69) | 1.34 (1.24-1.44) |
| *P* value for trend |  | < 0.001 | < 0.001 | < 0.001 |
| Social isolation |  |  |  |  |
| Index = 0 | 2733/604350 | Ref. | Ref. | Ref. |
| Index = 1 | 3299/584391 | 1.25 (1.19-1.31) | 1.19 (1.13-1.25) | 1.13 (1.07-1.19) |
| Index ≥ 2 | 1644/216724 | 1.68 (1.58-1.78) | 1.46 (1.37-1.55) | 1.26 (1.18-1.34) |
| *P* value for trend |  | < 0.001 | < 0.001 | < 0.001 |
| **Psychotic disorders** |  |  |  |  |
| Loneliness |  |  |  |  |
| Index = 0 | 139/927164 | Ref. | Ref. | Ref. |
| Index = 1 | 121/404998 | 1.99 (1.56-2.55) | 1.79 (1.40-2.29) | 1.73 (1.35-2.21) |
| Index = 2 | 42/114317 | 2.45 (1.74-3.47) | 2.07 (1.46-2.93) | 1.90 (1.34-2.70) |
| *P* value for trend |  | < 0.001 | < 0.001 | < 0.001 |
| Social isolation |  |  |  |  |
| Index = 0 | 80/618884 | Ref. | Ref. | Ref. |
| Index = 1 | 138/602042 | 1.78 (1.35-2.34) | 1.64 (1.24-2.16) | 1.59 (1.21-2.10) |
| Index ≥ 2 | 84/225552 | 2.89 (2.13-3.93) | 2.37 (1.74-3.23) | 2.21 (1.62-3.03) |
| *P* value for trend |  | < 0.001 | < 0.001 | < 0.001 |
| **Mood disorders** |  |  |  |  |
| Loneliness |  |  |  |  |
| Index = 0 | 4005/907666 | Ref. | Ref. | Ref. |
| Index = 1 | 3090/389156 | 1.81 (1.72-1.89) | 1.74 (1.66-1.82) | 1.67 (1.59-1.75) |
| Index = 2 | 1318/107340 | 2.80 (2.63-2.98) | 2.62 (2.46-2.79) | 2.38 (2.24-2.54) |
| *P* value for trend |  | < 0.001 | < 0.001 | < 0.001 |
| Social isolation |  |  |  |  |
| Index = 0 | 3110/603085 | Ref. | Ref. | Ref. |
| Index = 1 | 3604/584058 | 1.20 (1.14-1.26) | 1.14 (1.09-1.20) | 1.09 (1.04-1.15) |
| Index ≥ 2 | 1699/217020 | 1.52 (1.44-1.62) | 1.42 (1.33-1.50) | 1.30 (1.22-1.38) |
| *P* value for trend |  | < 0.001 | < 0.001 | < 0.001 |
| **Depression** |  |  |  |  |
| Loneliness |  |  |  |  |
| Index = 0 | 3906/908344 | Ref. | Ref. | Ref. |
| Index = 1 | 3018/389621 | 1.81 (1.72-1.90) | 1.74 (1.66-1.83) | 1.67 (1.59-1.75) |
| Index = 2 | 1302/107456 | 2.84 (2.67-3.02) | 2.66 (2.50-2.83) | 2.42 (2.27-2.57) |
| *P* value for trend |  | < 0.001 | < 0.001 | < 0.001 |
| Social isolation |  |  |  |  |
| Index = 0 | 3042/603582 | Ref. | Ref. | Ref. |
| Index = 1 | 3524/584547 | 1.20 (1.14-1.26) | 1.14 (1.09-1.20) | 1.10 (1.04-1.15) |
| Index ≥ 2 | 1660/217293 | 1.52 (1.43-1.62) | 1.42 (1.33-1.51) | 1.30 (1.22-1.38) |
| *P* value for trend |  | < 0.001 | < 0.001 | < 0.001 |
| **Anxiety disorders** |  |  |  |  |
| Loneliness |  |  |  |  |
| Index = 0 | 3210/914020 | Ref. | Ref. | Ref. |
| Index = 1 | 2147/396254 | 1.55 (1.47-1.64) | 1.50 (1.42-1.58) | 1.45 (1.38-1.54) |
| Index = 2 | 818/110810 | 2.12 (1.96-2.29) | 2.02 (1.87-2.18) | 1.88 (1.74-2.03) |
| *P* value for trend |  | < 0.001 | < 0.001 | < 0.001 |
| Social isolation |  |  |  |  |
| Index = 0 | 2399/608784 | Ref. | Ref. | Ref. |
| Index = 1 | 2670/591129 | 1.15 (1.09-1.22) | 1.10 (1.04-1.17) | 1.07 (1.01-1.13) |
| Index ≥ 2 | 1106/221170 | 1.28 (1.19-1.37) | 1.21 (1.13-1.30) | 1.14 (1.06-1.22) |
| *P* value for trend |  | < 0.001 | < 0.001 | < 0.001 |
| **PTSD** |  |  |  |  |
| Loneliness |  |  |  |  |
| Index = 0 | 67/927507 | Ref. | Ref. | Ref. |
| Index = 1 | 48/405376 | 1.64 (1.13-2.38) | 1.58 (1.09-2.29) | 1.48 (1.02-2.15) |
| Index = 2 | 24/114419 | 2.91 (1.83-4.64) | 2.58 (1.61-4.13) | 2.21 (1.37-3.55) |
| *P* value for trend |  | < 0.001 | < 0.001 | < 0.001 |
| Social isolation |  |  |  |  |
| Index = 0 | 48/619037 | Ref. | Ref. | Ref. |
| Index = 1 | 56/602433 | 1.20 (0.82-1.77) | 1.14 (0.77-1.67) | 1.09 (0.74-1.61) |
| Index ≥ 2 | 35/225833 | 2.02 (1.30-3.12) | 1.78 (1.15-2.77) | 1.61 (1.03-2.51) |
| *P* value for trend |  | 0.002 | 0.011 | 0.039 |
| **Behavioral syndromes** |  |  |  |  |
| Loneliness |  |  |  |  |
| Index = 0 | 122/927249 | Ref. | Ref. | Ref. |
| Index = 1 | 73/405274 | 1.37 (1.03-1.83) | 1.36 (1.02-1.82) | 1.34 (1.00-1.80) |
| Index = 2 | 27/114377 | 1.80 (1.18-2.73) | 1.79 (1.17-2.72) | 1.72 (1.12-2.62) |
| *P* value for trend |  | 0.002 | 0.002 | 0.004 |
| Social isolation |  |  |  |  |
| Index = 0 | 95/618832 | Ref. | Ref. | Ref. |
| Index = 1 | 87/602275 | 0.94 (0.71-1.26) | 0.97 (0.72-1.30) | 0.97 (0.72-1.30) |
| Index ≥ 2 | 40/225792 | 1.16 (0.80-1.68) | 1.11 (0.76-1.61) | 1.12 (0.76-1.63) |
| *P* value for trend |  | 0.512 | 0.650 | 0.626 |

Abbreviations: CI, Confidence interval; HR, Hazard ratio; PTSD: post-traumatic stress disorder.

Model 1: without adjustment.

Model 2: adjusted for age, sex, ethnicity, Townsend deprivation index, and education.

Model 3: adjusted for age, sex, ethnicity, Townsend deprivation index, education, diet, smoking status, alcohol consumption, exercise, sleep duration, and the number of chronic diseases.
